# Supplementary material for: Self-reporting photodynamic nanobody conjugate for precise and sustainable large-volume tumor treatment
Source: Nat Commun. 2024 Aug 13;15:6935. doi: 10.1038/s41467-024-51253-5 (PMC11322375; doi:10.1038/s41467-024-51253-5)
Supplement: Supplementary file 1 — Supplementary Information [file 41467_2024_51253_MOESM1_ESM.pdf]

Supplementary Information

Self-Reporting Photodynamic Nanobody Conjugate for Precise and Sustainable Large-Volume Tumor Treatment

Yingchao Chen[1], *Tao Xiong*[1], *Qiang Peng*[1], *Jianjun Du*[1], *Wen Sun*[1], *Jiangli Fan*[1]*, *Xiaojun Peng*[1]

[1]State Key Laboratory of Fine Chemicals, Frontiers Science Center for Smart Materials Oriented Chemical Engineering, Dalian University of Technology, No. 2 Linggong Road, Dalian 116024, China
E-mail: fanjl@dlut.edu.cn

Instruments

^{1}H nuclear magnetic resonance (^{1}H NMR) and ^{13}C NMR spectra of all compounds were detected by Bruker Avance II 500 MHz spectrometer. The mass spectrometric (MS) data were obtained with electrospray ionization (EIS)-high resolution mass spectrometry (HRMS) instruments. Absorption spectra were performed with a Lambda 60 UV-visible (UV-Vis) spectrophotometer (PerkinElmer, USA). And fluorescence spectra were tested in a VAEIAN CARY Eclipse fluorescence spectrophotometer (Serial No. FL0812-M018). The bind affinity of Nbs was evaluated by ForteBio Octet RED 96e (Sartorius, Germany). The cell imaging experiments were performed on confocal laser scanning microscope (CLSM, Olympus FV3000, Japan). Cytotoxicity assays were analyzed by Thermo Varioskan™ LUX multifunctional microplate reader (USA). The *in vivo* imaging experiments were carried out on NightOWL II LB983 small animal *in vivo* imaging system (Germany). Flow cytometry was analyzed by Attune NxT Acoustic Focusing Cytometer.

Synthesis

Synthesis of compound 1

1-naphthylamine (1 g, 7 mmol) and 3-bromopropionamide (2.1 g, 14 mmol) were dissolved in 20 mL 2-methoxyethanol, then K_2CO_3 (1 g, 7 mmol) and CuI (190 mg, 1 mmol) was added into the mixture, the reaction was stirred under reflux for 12 h. After rotary evaporator, the crude product was purified by column chromatography (petroleum ether: DCM= 10:1 v/v) to give an oil product compound 1 (1.26 g, 6.3 mmol, 90%). HRMS (ESI): m/z for $C_{13}H_{17}N_2^+$ ($[M+H]^+$): calc. 201.1386; found 201.1392.

Synthesis of compound MNB

After $Al_2(SO_4)_3$ (2 g, 6 mmol) was dissolved in 10 mL water, 4-morpholinoaniline (1 g, 6 mmol), $Na_2S_2O_3$ (2 g, 12 mmol) and $ZnCl_2$ (1 g, 6 mmol) were added into this solution one by one, then $K_2Cr_2O_7$ (600 mg, 2 mmol) was slowly added into mixture under ice-

bath. The reaction was stirred under 0-4 °C for 2 h. The gray solid compound 2 was gathered after filtration and washes of acetone. To prepare the compound MNH_2, compound 1 (400 mg, 2 mmol) and compound 2 (700 mg, 2.4 mmol) were dissolved in dimethyl sulfoxide (20 mL), and then $K_2Cr_2O_7$ (600 mg, 2 mmol) was slowly poured into flask under mixture. After 20 min reaction, the purple mixture was dropped into methanol (200 mL), the pH was adjusted using HCl (8 mL, 1M), then blue solution was obtained after 45 min reaction. After rotary evaporator, the crude product was purified by column chromatography (CH_2Cl_2:CH_3OH= 20:1 v/v) to give a blue solid (650 mg, 73%). HRMS (ESI): m/z for $C_{23}H_{25}N_4OS^+$ ($[M]^+$): calc. 405.1744; found 405.1747.

Synthesis of compound 3

4-nitrophenylhydrazine (2 g, 13mmol), ethyl acetoacetate (2 g, 15mmol) and sodium acetate (1 g, 13mmol) were added into 30 mL ethanol, and the mixture was stirred at 78 °C under N_2 atmosphere for 12h. The solvent was then evaporated under reduced pressure and the residue was used for next step reaction. The residue and $SnCl_2$ (5 g, 26 mmol) were dissolved in 100 mL methanol and the mixture was stirred at 64 °C for 10 h. After removal of the solvent under reduced pressure, the residue was subjected to silica gel chromatography with CH_2Cl_2/CH_3OH (10/1, *v*/*v*) as the eluent. Finally, compound 3 as brown gummy liquid (46 mg, 60%) was afforded. HRMS (ESI): m/z for $C_{10}H_{12}N_3O^+$ ($[M+H]^+$): calc. 190.0975; found 190.0977.

Synthesis of compound 4

Compound 3 (100 mg, 0.53 mmol), HATU (402 mg, 1 mmol) and TMP (128 mg, 1 mmol) were dissolved in 3 mL DMF and stirred under room temperature. Then 2,2'-[propane-2,2-diylbis(thio)]diacetic acid (240 mg, 1mmol) was added into the mixture and the reaction was stirred room temperature for 8h. The solvent was then evaporated under reduced pressure and the residue purified by silica gel column chromatography by using CH_2Cl_2/CH_3OH 5:1 (*v*/*v*) as eluent, affording compound 4 (125 mg, 60%) as colorless gummy liquid. HRMS (ESI): m/z for $C_{17}H_{22}N_3O_4S_2^+$ ($[M+H]^+$): calc. 396.1046; found 396.1048.

Synthesis of MNB-Pyra

Compound 4 (100 mg, 0.25 mmol), HATU (190 mg, 0.5 mmol) and TMP (60 mg, 0.5 mmol) were dissolved in 3 mL DMF and stirred under room temperature. Then MNH_2 (220 mg, 0.5mmol) was added into the mixture and the reaction was stirred room temperature for 8h. The solvent was then evaporated under reduced pressure and the residue purified by silica gel column chromatography by using CH_2Cl_2/CH_3OH 10:1 (*v*/*v*) as eluent, affording MNB-Pyra (150 mg, 80%) as blue solid. HRMS (ESI): m/z for $C_{40}H_{44}N_7O_4S_3^+$ ($[M]^+$): calc. 782.2611; found 782.2601. m/z for $C_{40}H_{45}N_7O_4S_3^{2+}$ ($[M+H]^{2+}$): calc. 396.6342; found 396.6336. 1H NMR (500 MHz, DMSO-d6) δ 10.17 (s, 1H), 10.05 (s, 1H), 9.03 (d, *J* = 8.3 Hz, 1H), 8.52 (d, *J* = 7.7 Hz, 1H), 8.25 – 8.21 (m, 1H), 8.05 (d, *J* = 9.3 Hz, 1H), 8.00 – 7.96 (m, 1H), 7.92 (t, *J* = 7.3 Hz, 1H), 7.65 (s, 1H), 7.58 (s, 2H), 7.56 (s, 3H), 5.32 (s, 1H), 3.79 – 3.75 (t, *J* = 4.2 Hz, 4H), 3.75 – 3.68 (s, 4H), 3.49 (t, *J* = 5 Hz, 4H), 3.28 – 3.24 (t, *J* = 11.6 Hz, 2H), 2.07 (s, 2H), 2.04 – 1.97 (m, 2H), 1.95 – 1.91 (t, *J* = 6.5 Hz, 2H), 1.59 (s, 6H), 1.24 (s, 3H). ^{13}C NMR (151 MHz, MeOD) δ 159.28, 157.64, 154.20, 152.51, 141.55, 136.71, 136.26, 133.59, 133.18,

131.58, 130.55, 130.41, 125.58, 124.73, 122.86, 121.48, 120.27, 120.06, 118.97, 117.30, 106.22, 102.92, 66.21, 57.30, 54.39, 46.95, 46.53, 41.83, 36.92, 35.69, 34.66, 31.82, 29.81, 29.54, 29.24, 28.32, 22.54, 16.24, 13.58, 8.29.

Synthesis of MNB-Pyra dimer

MNB-Pyra (50 mg, 0.06 mmol) and isobutyraldehyde (2.16 mg, 0.03 mmol) were dissolved in 2 mL DMF and the mixture was stirred at room temperature for 24 h. The solvent was then evaporated under reduced pressure and the residue purified by silica gel column chromatography by using CH_2Cl_2/CH_3OH 8:1 (*v*/*v*) as eluent, affording MNB-Pyra dimer (72 mg, 70%) as blue solid. HRMS (ESI): m/z for $C_{84}H_{94}N_{14}O_8S_6^{2+}$ ($[M]^{2+}$): calc. 1618.5692; found 1618.5286. ^{1}H NMR (500 MHz, DMSO) δ 10.22 (s, 2H), 9.93 (s, 2H), 8.86 (d, *J* = 7.9 Hz, 2H), 8.39 (d, *J* = 7.9 Hz, 2H), 8.25 (d, *J* = 5.5 Hz, 2H), 7.85 (dt, *J* = 15.0, 7.1 Hz, 6H), 7.63 (s, 2H), 7.56 (s, 4H), 7.50 (s, 2H), 7.42 (d, *J* = 9.9 Hz, 2H), 7.34 (d, *J* = 14.2 Hz, 2H), 3.78 – 3.70 (t, 7.5 Hz, 4H), 3.67 – 3.56 (t, 4.3 Hz, 12H), 3.50 – 3.47 (t, 5.4 Hz, 4H), 3.27 – 3.24 (t, 4.7 Hz, 4H), 3.17 (s, 2H), 2.96 (d, *J* = 11.0 Hz, 2H), 2.18 (s, 6H), 2.04 – 1.97 (m, 1H), 1.92 (dt, *J* = 13.2, 6.5 Hz, 4H), 1.59 (s, 12H), 1.49 – 1.41 (m, 1H), 1.23 (s, 6H), 0.81 (d, *J* = 6.1 Hz, 6H). ^{13}C NMR (126 MHz, DMSO) δ 169.01, 167.51, 153.56, 151.97, 145.69, 140.50, 136.14, 135.00, 132.92, 132.22, 131.43, 130.15, 129.93, 124.96, 124.26, 123.14, 120.89, 119.51, 119.35, 117.29, 106.40, 103.69, 73.86, 65.71, 59.99, 57.98, 56.42, 48.56, 46.63, 41.99, 36.86, 36.63, 35.37, 35.08, 34.31, 30.21, 28.62, 28.28, 28.03, 21.41, 13.90, 11.50.

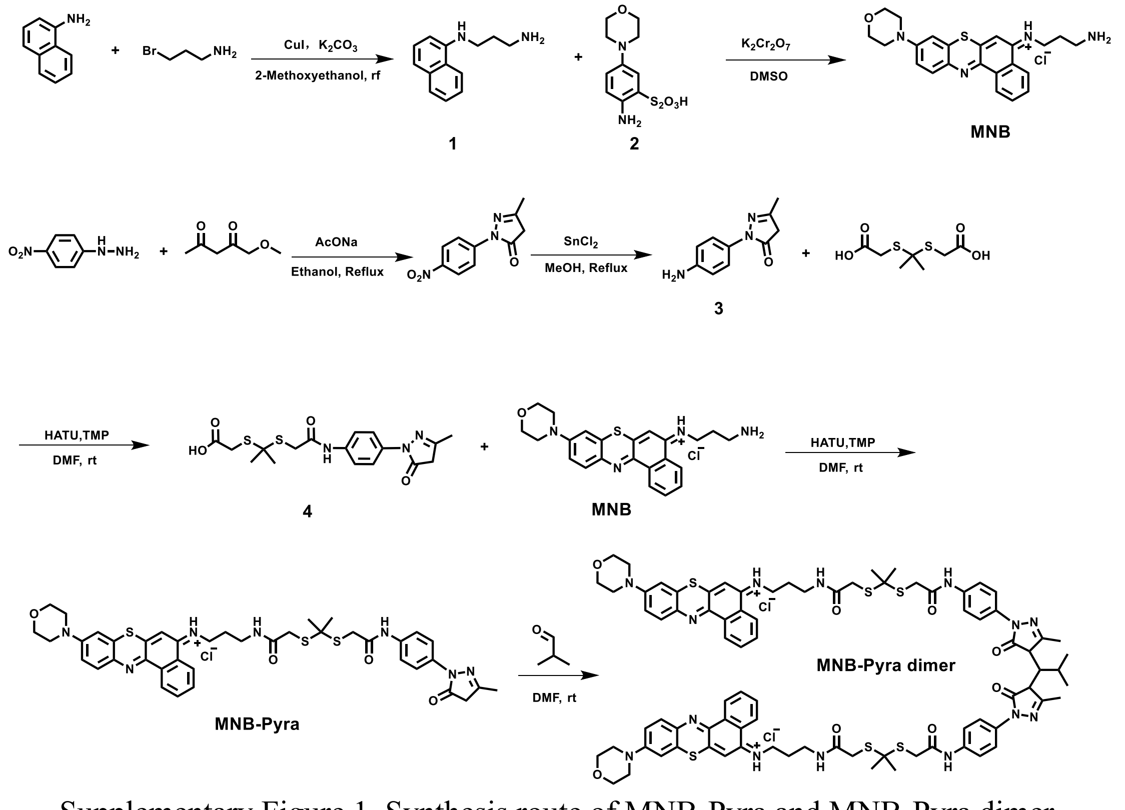

Supplementary Figure 1. Synthesis route of MNB-Pyra and MNB-Pyra dimer.

Compound	Φ_f in CH_3CN	Φ_f in H_2O
MNB-Pyra	12.4%	4.6%
MNB-Pyra dimer	4.1%	0.9%
MNB-Pyra Nbs	/	0.8%

Supplementary Table 1. Absolute fluorescence quantum yield of MNB-Pyra, MNB-Pyra dimer and MNB-Pyra Nbs in organic different solutions.

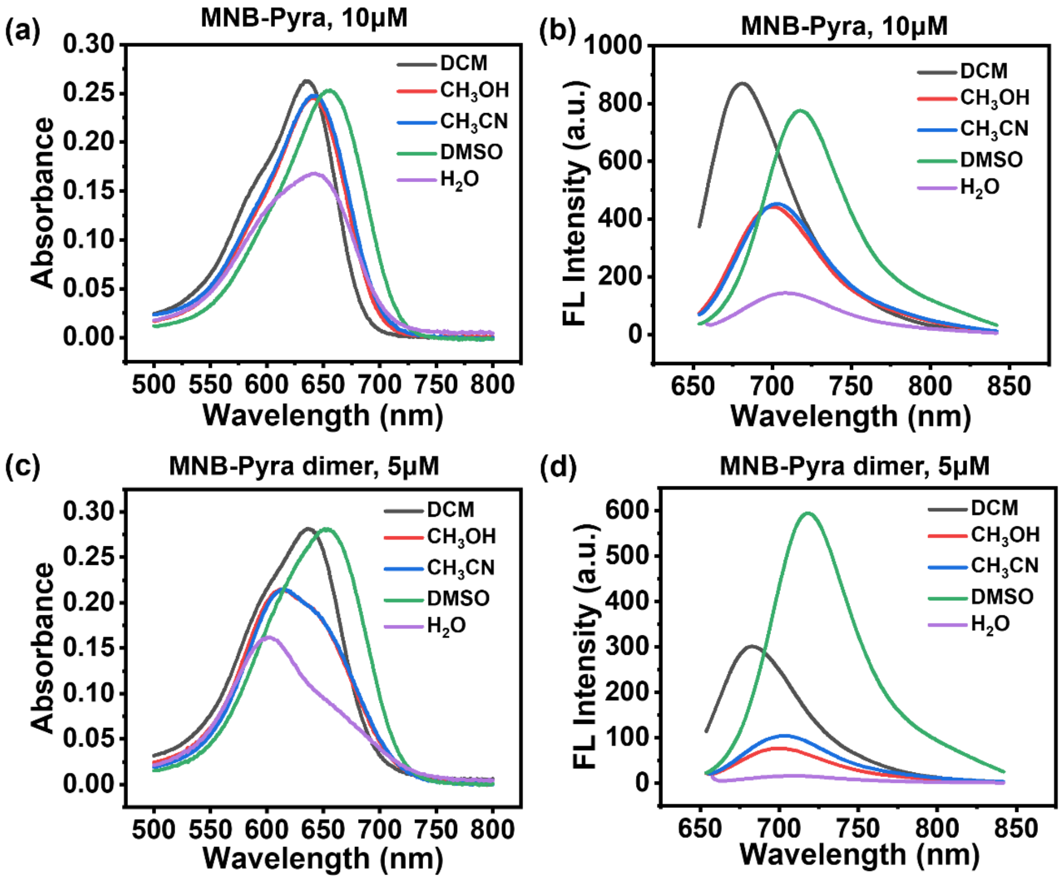

Supplementary Figure 2. (a) Absorption and (b) fluorescence spectrum of MNB-Pyra (10 μM) in different solvents. (c) Absorption and (d) fluorescence spectrum of MNB-Pyra dimer (5 μM) in different solvents.

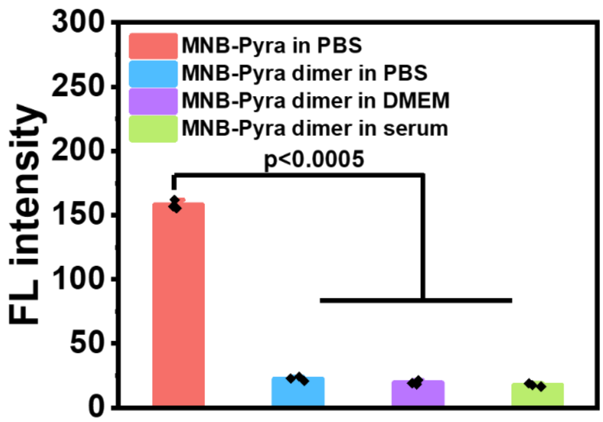

Supplementary Figure 3. Fluorescence intensity of MNB-Pyra and MNB-Pyra dimer in biological solutions, n = 3 experimental replicates, data are shown as mean ± SD. Statistical significance was calculated via one tailed Student's t test. $P < 0.05$ is considered to be statistically significant. Source data are provided as a Source Data file.

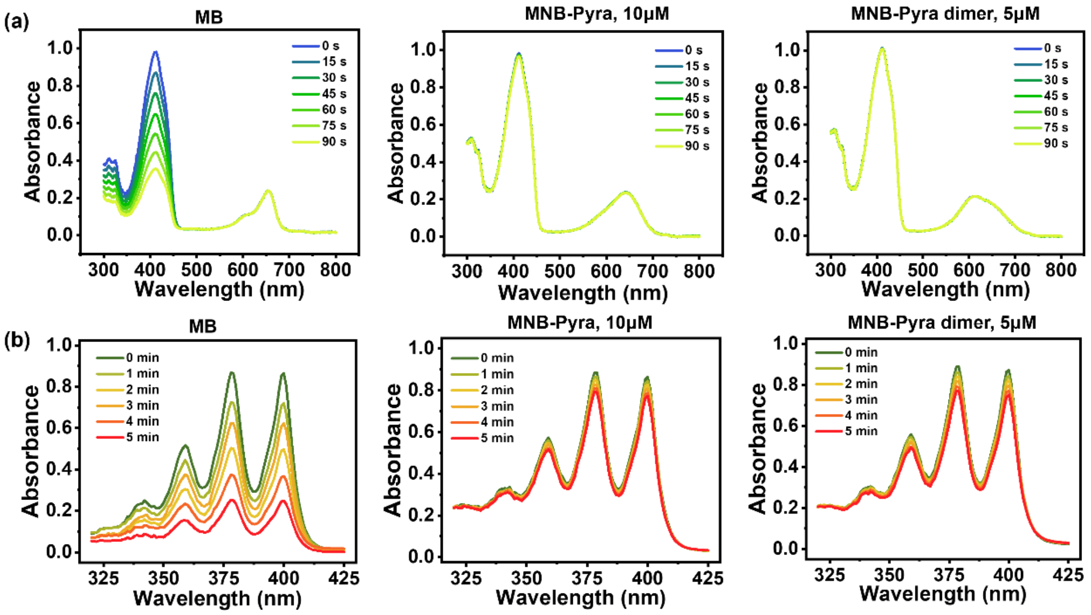

Supplementary Figure 4. (a) Detection of $^1O_2$ generation by MNB-Pyra and MNB-Pyra dimer using DPBF probe in MeOH solution. (b) Detection of $^1O_2$ generation by MNB-Pyra and MNB-Pyra dimer using ABDA probe in aqueous solution.

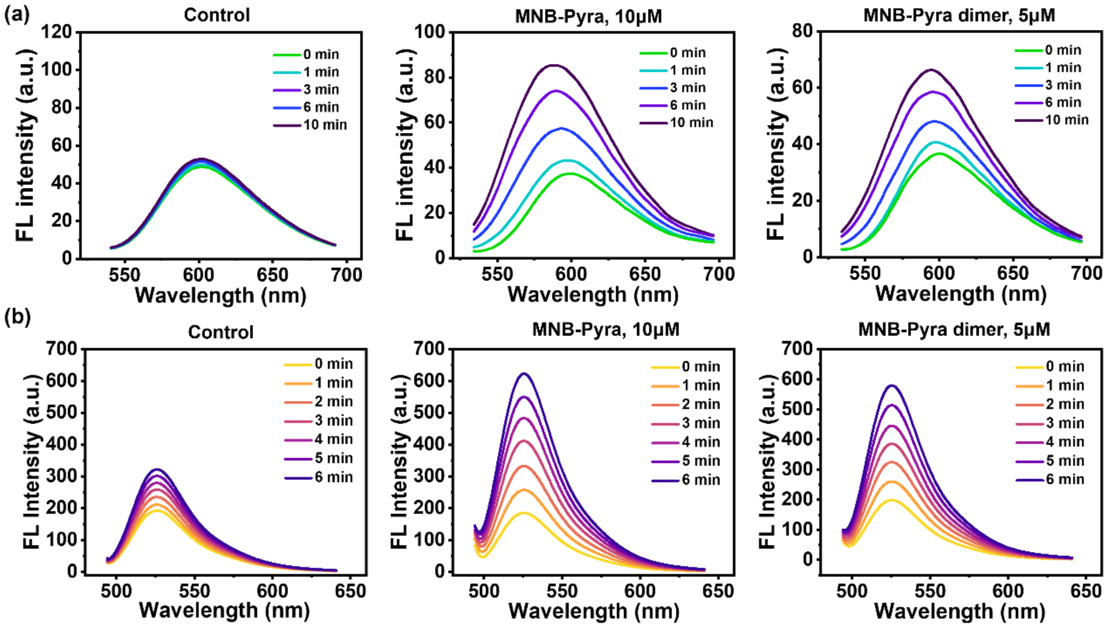

Supplementary Figure 5. (a) $O_2^{-\bullet}$ generation of MNB-Pyra and MNB-Pyra dimer detected by DHE probe with ctDNA in PBS solution. (b) $O_2^{-\bullet}$ generation of MNB-Pyra and MNB-Pyra dimer detected by DHR123 probe in PBS solution.

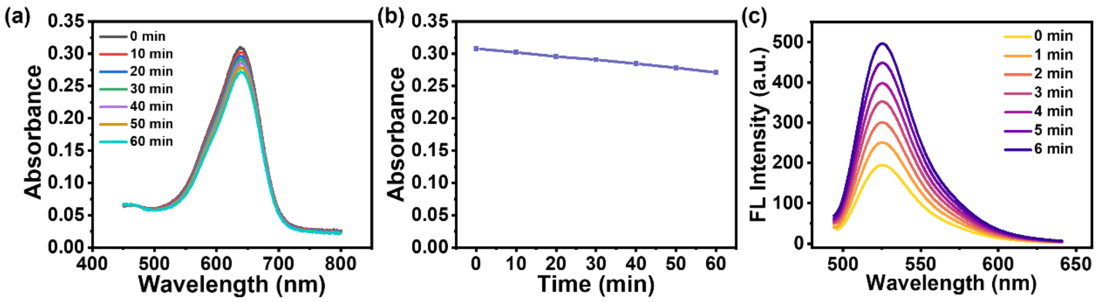

Supplementary Figure 6. (a) and (b) Photostability validation of **MNB-Pyra** (10 μM) under light irradiation (630 nm, 50 mW/cm^2) for 60 min. (c) ROS generation of **MNB-Pyra** (10 μM) detected by DHR123 probe after the irradiation (630 nm, 50 mW/cm^2) for 60 min.

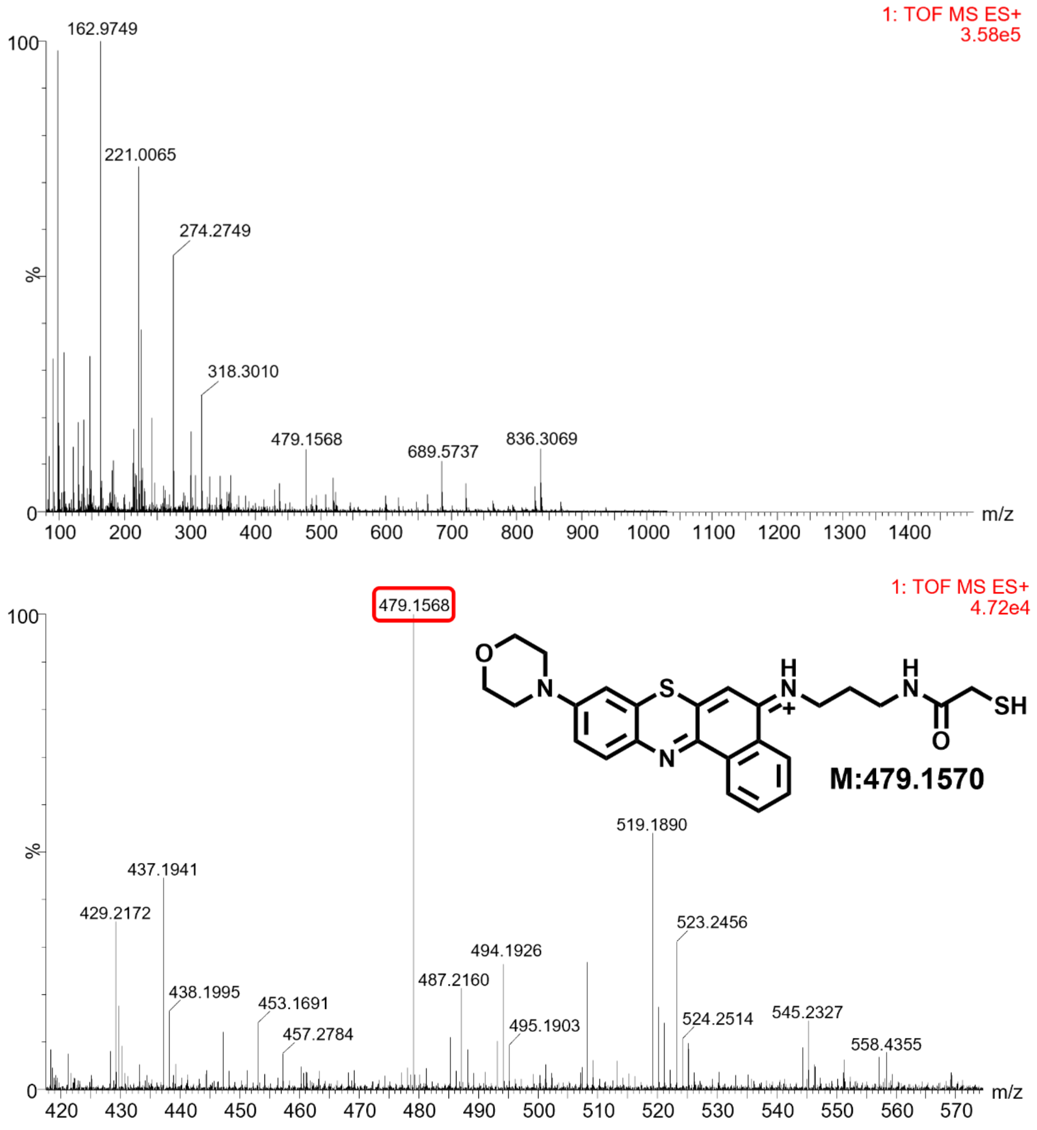

Supplementary Figure 7. HRMS analysis of cleavaed product after fluorescence recovery in MNB-Pyra dimer.

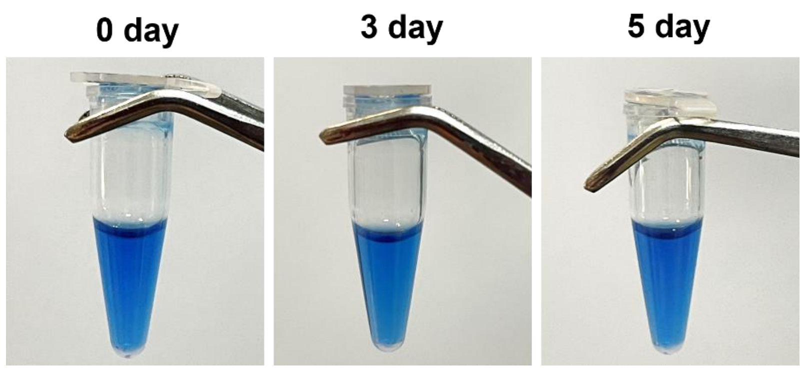

Supplementary Figure 8. Stability of MNB-Pyra Nbs (0.5 mM, 100 μL) in aqueous solution after being placed at 4°C for 5 days.

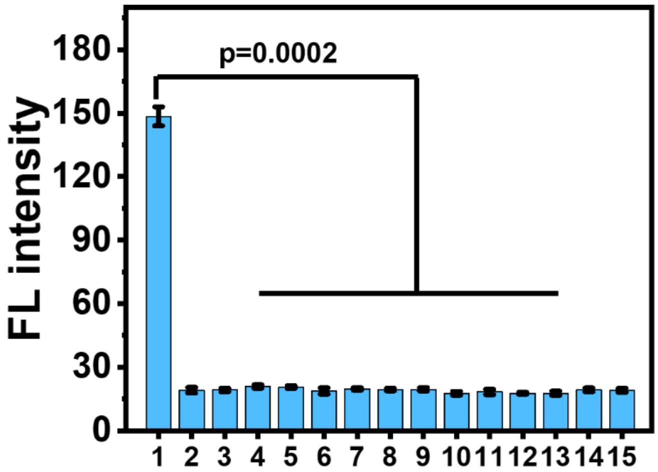

Supplementary Figure 9. A. Fluorescence of MNB-Pyra in PBS solution. Fluorescence intensity of MNB-Pyra Nbs in the presence of enzymes/other biomolecules (B: aminopeptidases, 50 ng/mL; C: γ-glutamyltransferase, 50 mU/mL; D: alkaline phosphatase, 50 ng/mL; E: nitroreductase, 10 μg/mL; F: esterase, 50 mU/mL; G: glutathione; H: cysteine; I: tyrosine; J: glycine; K: glutamate; L: arginine; M: tryptophan; N: aspartic acid; O: serine; 1 mM), n = 3 experimental replicates, data are shown as mean ± SD. Statistical significance was calculated via one tailed Student's t test. $P < 0.05$ is considered to be statistically significant. Source data are provided as a Source Data file.

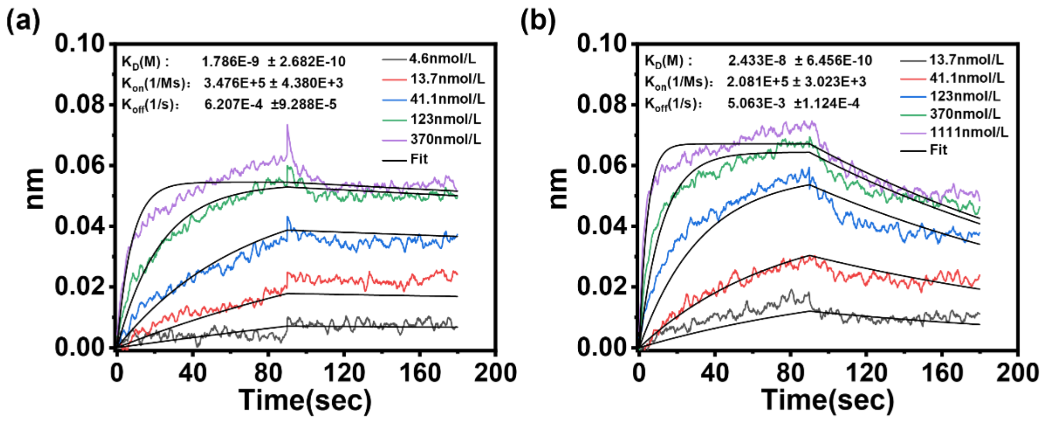

Supplementary Figure 10. Binding affinity evaluation of (a) 7D12-fGly Nbs and (b) MNB-Pyra Nbs with biolayer interferometry. Source data are provided as a Source Data file.

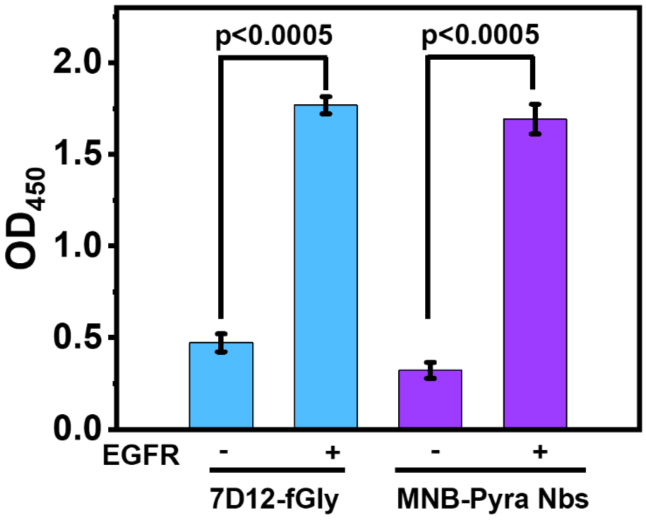

Supplementary Figure 11. Evaluating the cell binding of MNB-Pyra Nbs using cell-based ELISA. A431 cells were used as the EGFR positive cells, and NIH-3T3 cells were used as the negative control (n = 5 experimental replicates). Statistical significance was calculated via one tailed Student's t test. $P < 0.05$ is considered to be statistically significant. Data are shown as mean ± SD. Source data are provided as a Source Data file.

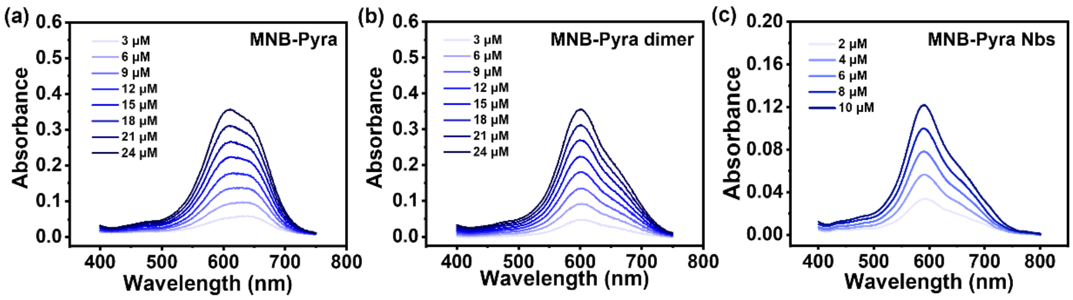

Supplementary Figure 12. Concentration-dependent (3-24 μM) absorbance spectra of (a) MNB-Pyra and (b) MNB-Pyra dimer in water. (c) Concentration-dependent (2-10 μM) trace absorbance spectra of MNB-Pyra Nbs. Source data are provided as a Source Data file.

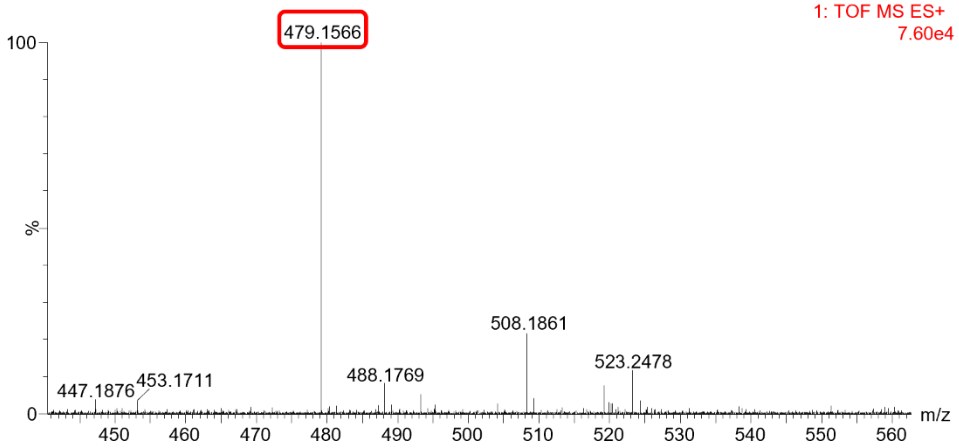

Supplementary Figure 13. Detection of cleavaed product after fluorescence recovery in MNB-Pyra Nbs.

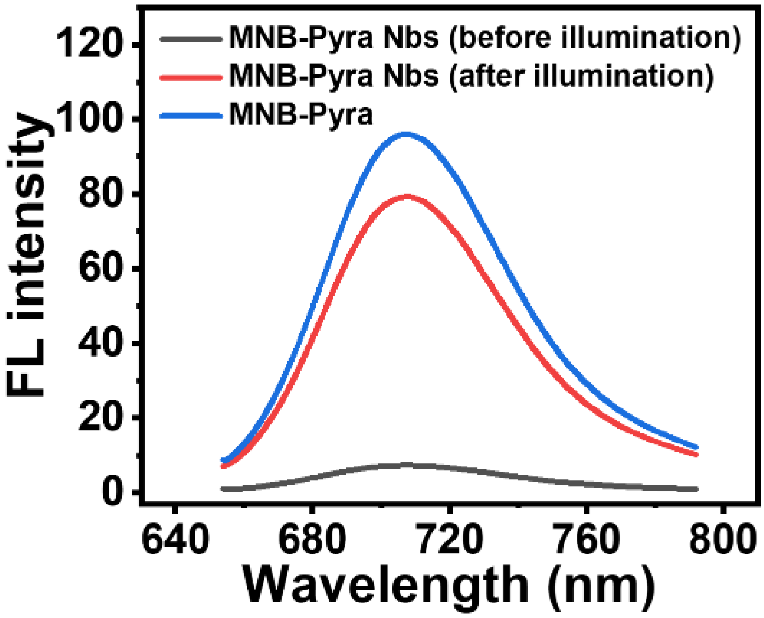

Supplementary Figure 14. Absorbance of MNB-Pyra Nbs (5 μM) after light irradiation in DMEM compared with that of MNB-Pyra (10 μM) to calculate the release efficiency of monomer photosensitizers. Source data are provided as a Source Data file.

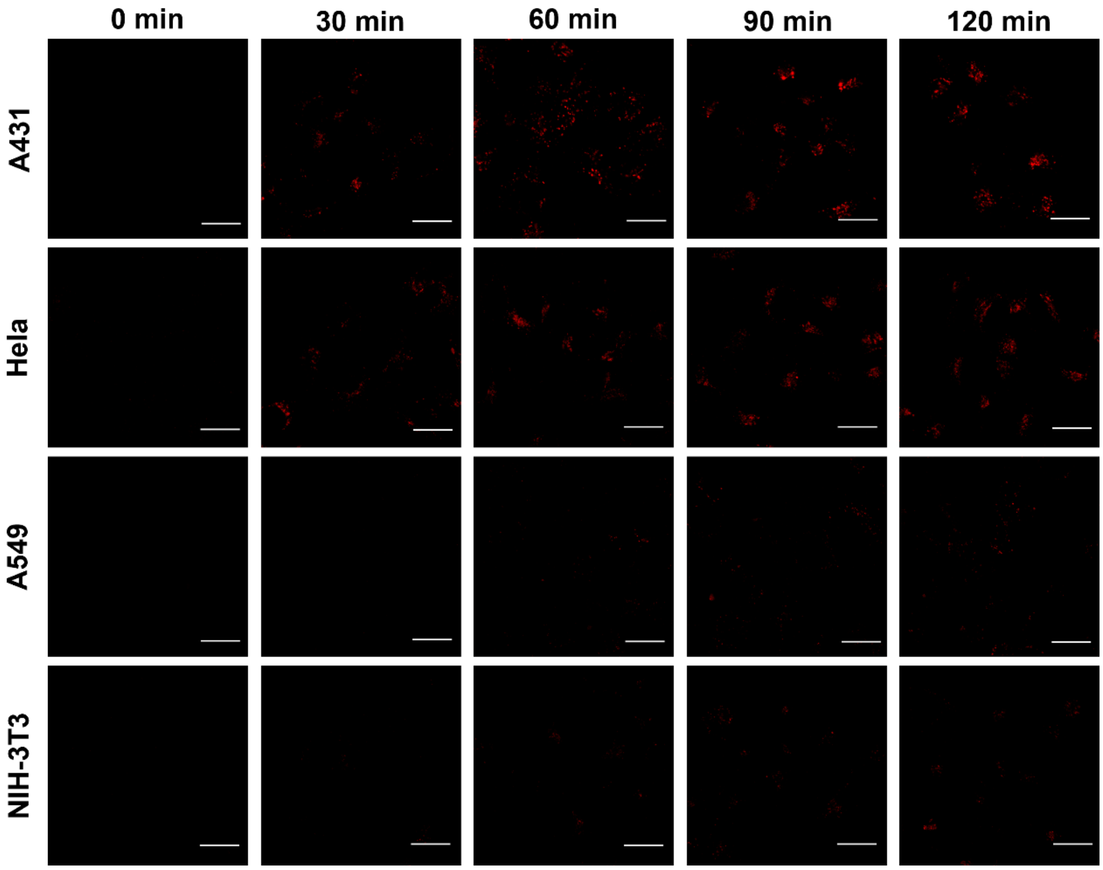

Supplementary Figure 15. Imaging of MNB-Pyra Nbs (0.2 μM) in different cells. Scale bar = 20 μm.

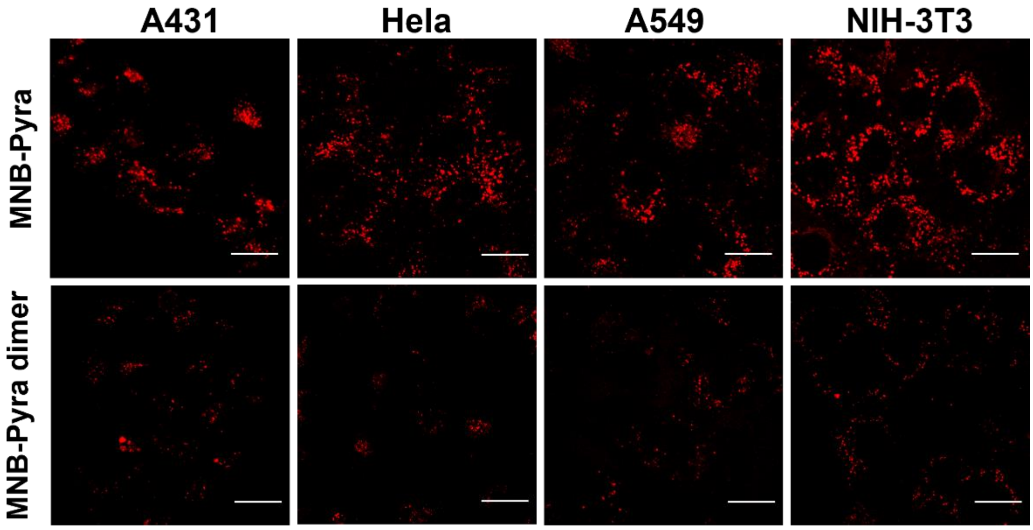

Supplementary Figure 16. Imaging of MNB-Pyra and MNB-Pyra dimer in different cells. Scale bar = 20 μm.

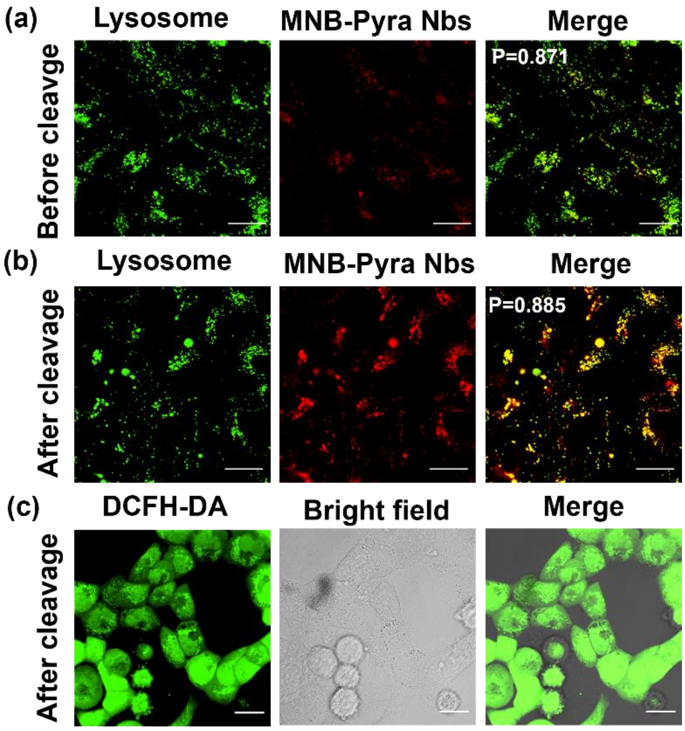

Supplementary Figure 17. (a) Co-localization of MNB-Pyra Nbs with lysosomes after incubating MNB-Pyra Nbs with A431 cells for 2 h. (b) Co-localization of released photosensitizers in A431 cells. (c) ROS detection in A431 cells with fluorescence probe DCFH-DA (E_x: 488 nm, E_m: 500-550 nm) after the nanobody conjugate cutting off. Scale bar = 20 μm.

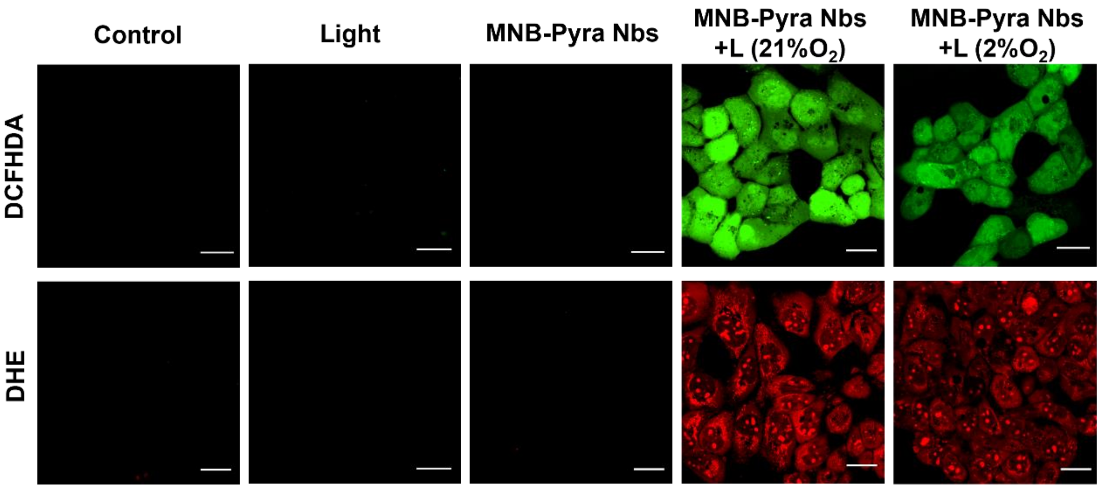

Supplementary Figure 18. ROS generating detection of MNB-Pyra Nbs in A431 cells under normoxia (21% O_2) and hypoxia (2% O_2) conditions with fluorescence probe DCFH-DA (E_x: 488 nm, E_m: 500-550 nm) and DHE (E_x: 560 nm, E_m: 600-630nm). Scale bar = 20 μm.

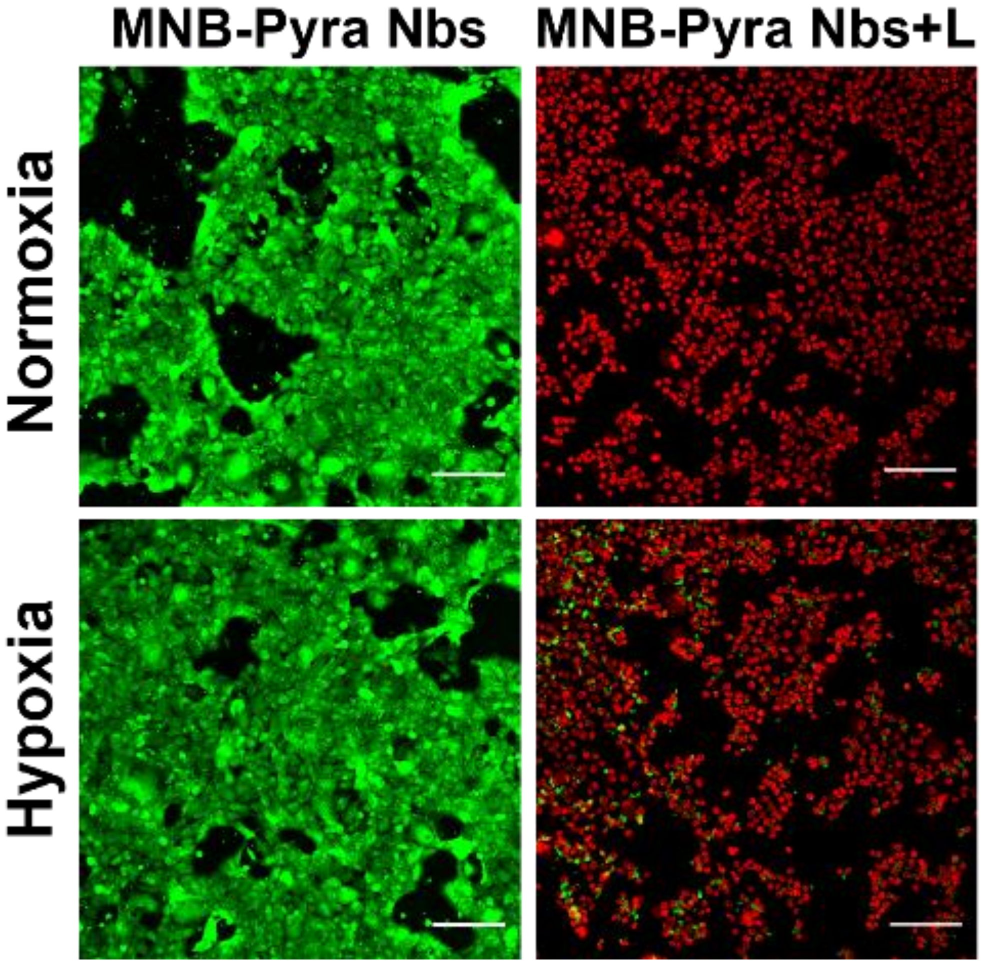

Supplementary Figure 19. Live/death cells imaging of A431 cells with PDT treatment under normoxia or hypoxia conditions using Calcien-AM/PI kit. Calcein-AM: E_x: 488 nm, E_m: 490–520 nm; PI: E_x: 488 nm, E_m: 600–700 nm. Scale bar = 200 µm.

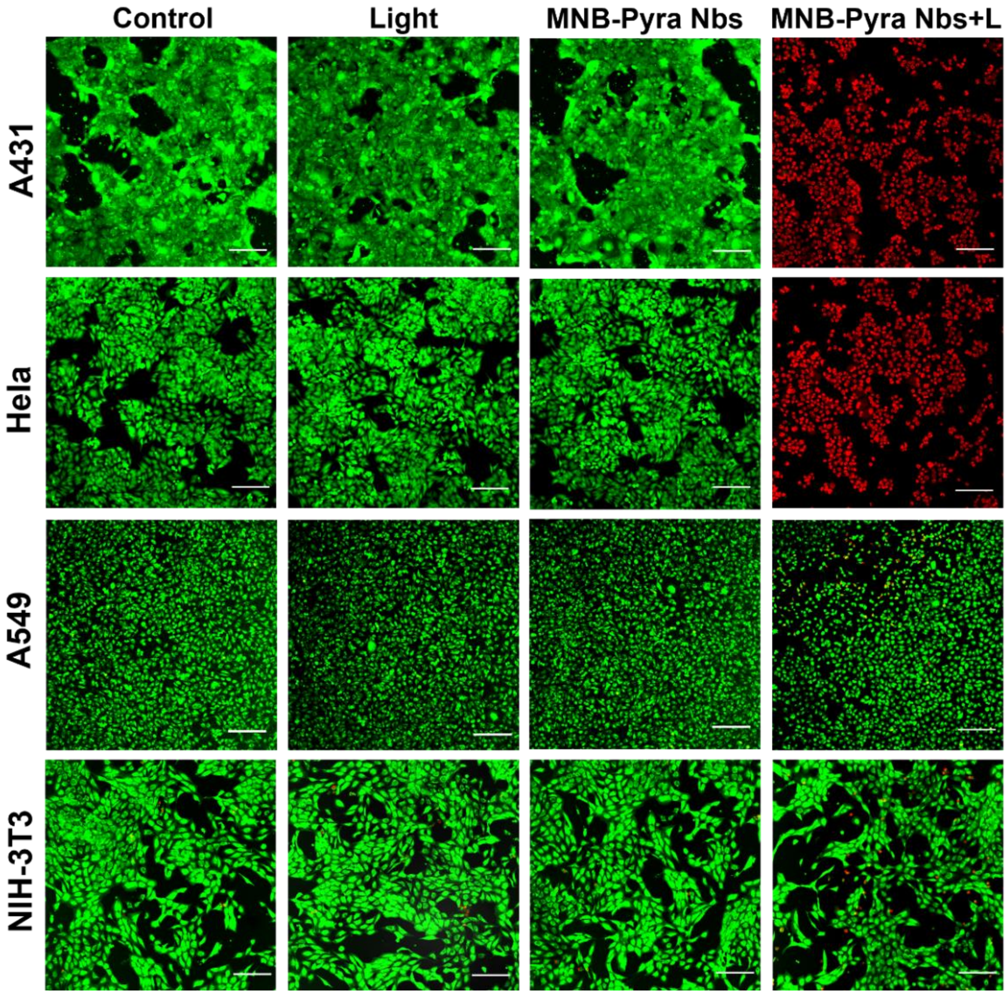

Supplementary Figure 20. Live/death cells imaging of different cells with or without PDT treatment (630 nm, 30 mW /cm^2, 20 min) using Calcien-AM/PI kit. Calcein-AM: E_x: 488 nm, E_m: 490–520 nm; PI: E_x: 488 nm, E_m: 600–700 nm. Scale bar = 200 μm.

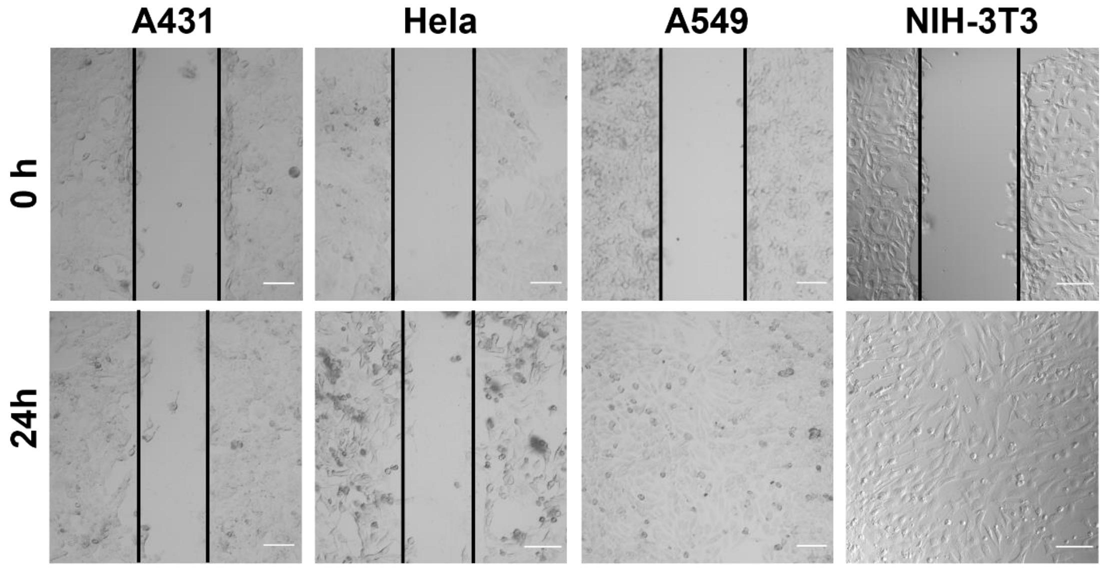

Supplementary Figure 21. Wound healing assay for comparing of recovery rate using MNB-Pyra Nbs in different cells. Scale bar = 200 μm.

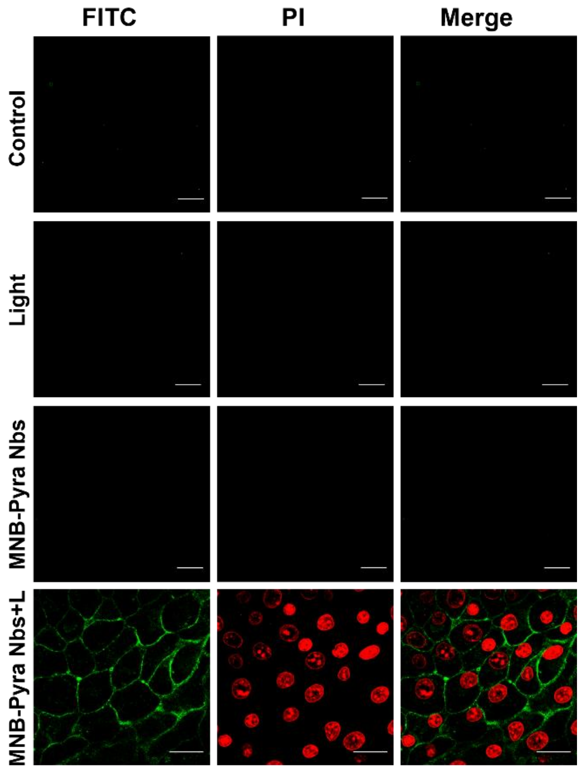

Supplementary Figure 22. Apoptosis imaging of A431 cells using Annexin V-FITC (E_x: 488 nm, E_m: 490-530 nm) and PI (E_x: 488 nm, E_m: 600-700 nm) kit. Scale bar = 20 μm.

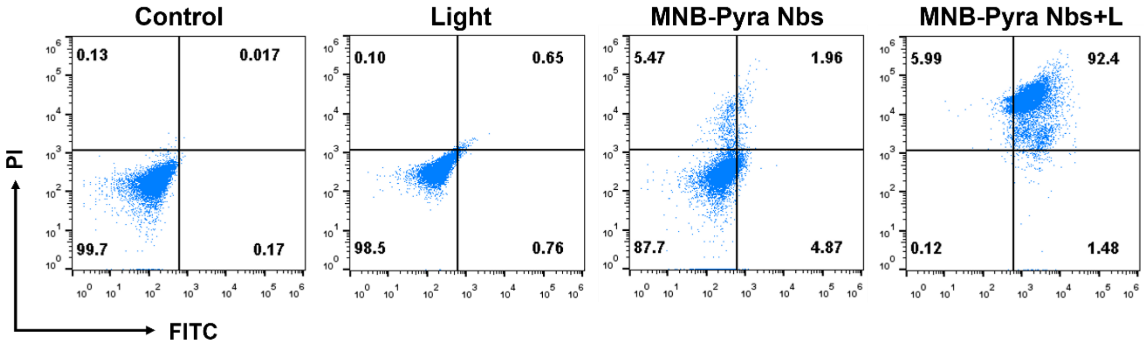

Supplementary Figure 23. Apoptosis detection using flow cytometry in A431 cells. The experiment was repeated three times with similar results.

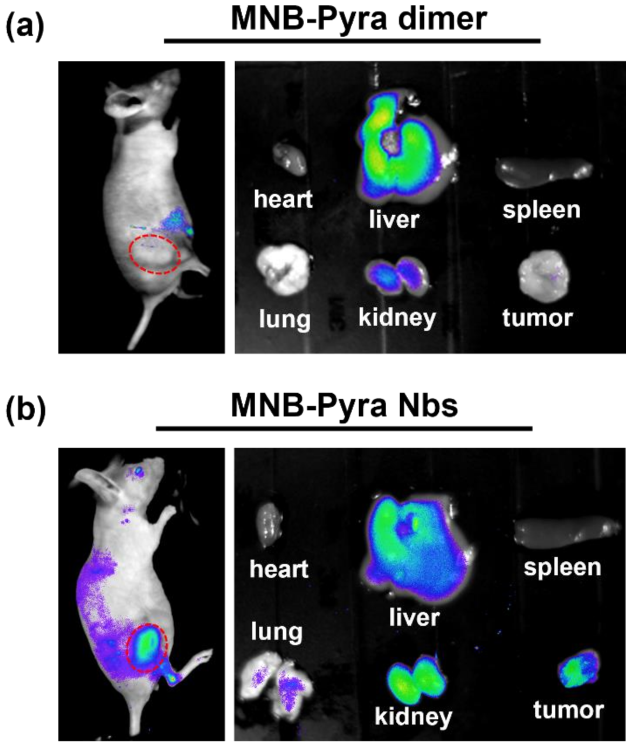

Supplementary Figure 24. (a) MNB-Pyra dime and (b) MNB-Pyra Nbs for in vivo imaging at 4 h postinjection.

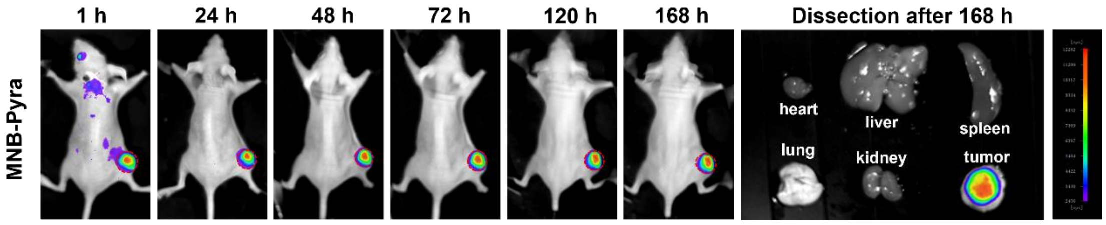

Supplementary Figure 25. Real-time tracking of MNB-Pyra in vivo after intratumorally injection.

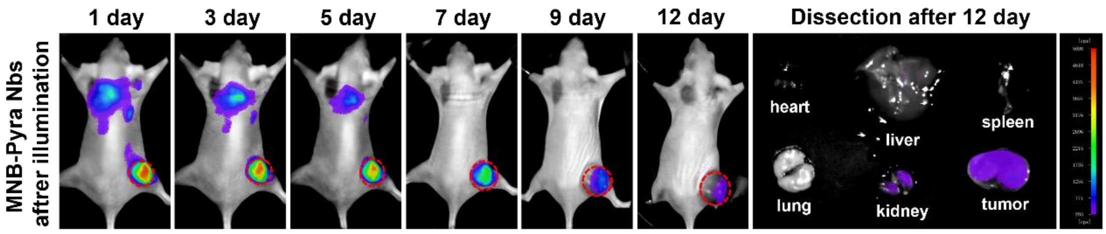

Supplementary Figure 26. Monitoring the clearance of the photosensitizers after released by light application.

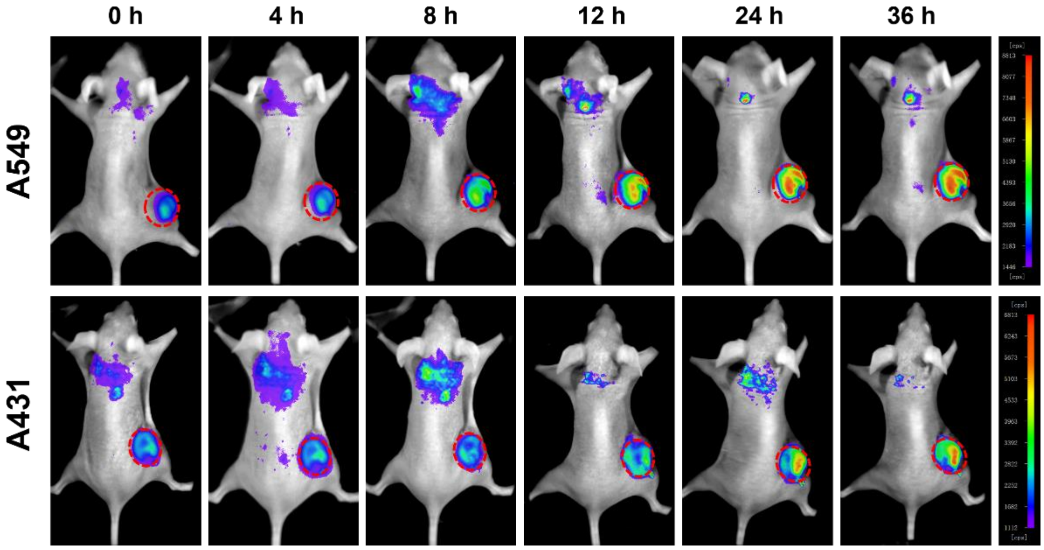

Supplementary Figure 27. Fluorescence observation after intratumorally injection of MNB-Pyra dimer (0.3 mM, 100 μL) in A549 and A431 tumor models.

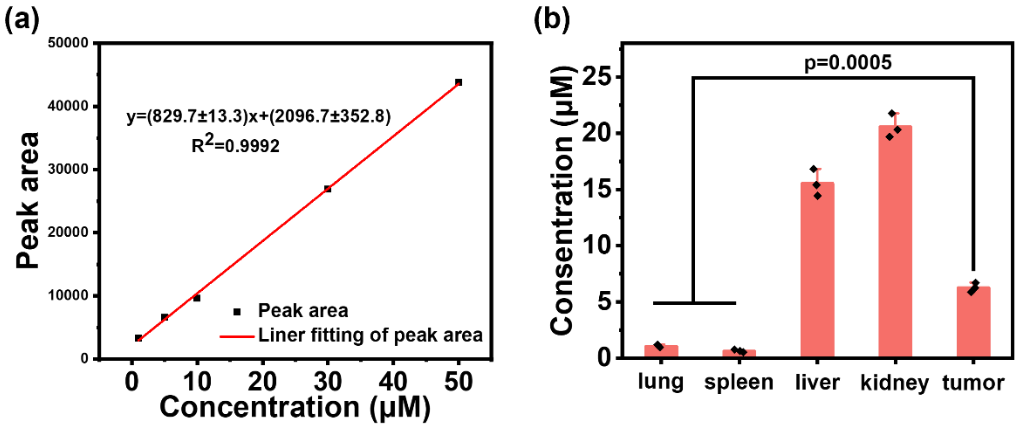

Supplementary Figure 28. (a) Standard curve of MNB-Pyra Nbs (1,5,10,30,50 μM) obtained by HPLC. (b) Drug concentrations in organs and tumor 4 hours after intravenous injection of MNB-Pyra Nbs (0.5 mM, 100 μL) in mice (n = 3 mice). Data are shown as mean ± SD. Statistical significance was calculated via one tailed Student's t test. $P < 0.05$ is considered to be statistically significant. Source data are provided as a Source Data file.

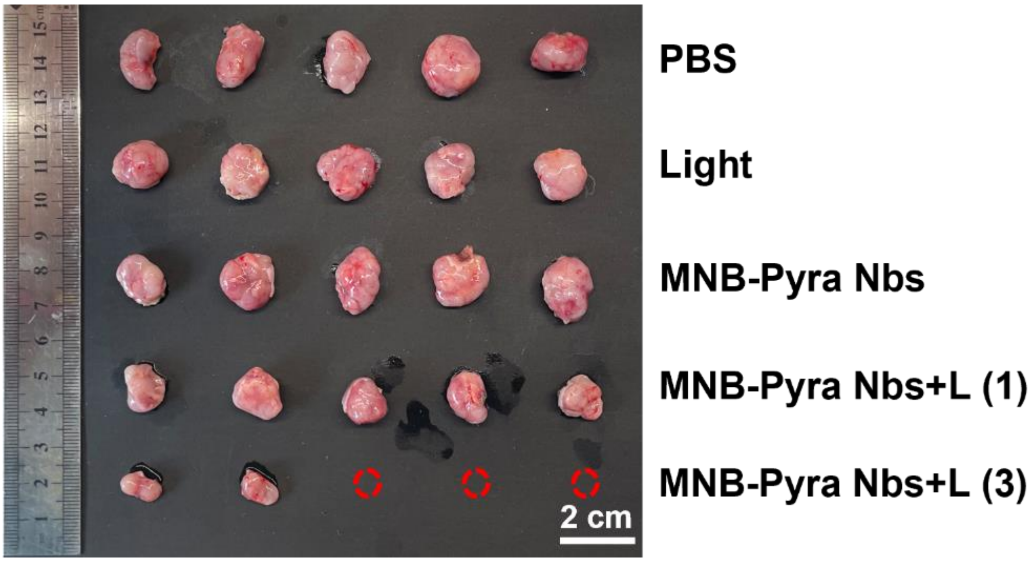

Supplementary Figure 29. Tumor picture at the end of experiment in different groups in A431 tumor bearing model. Scale bar = 2 cm.

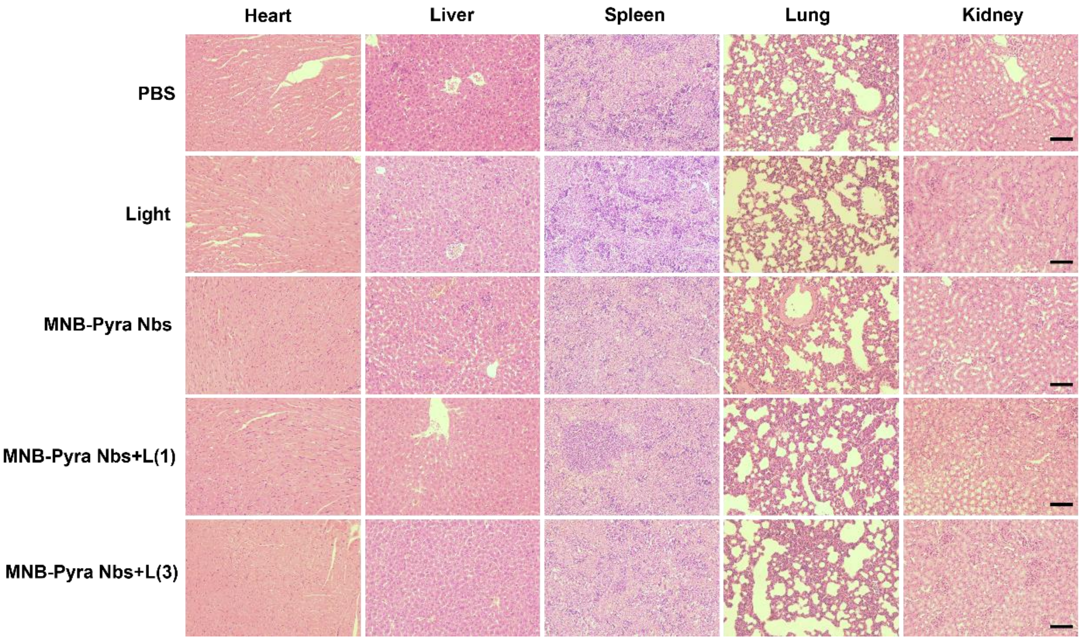

Supplementary Figure 30. H&E analysis of the major organs (heart, liver, spleen, lung, and kidney) tissues collected from mice in the different groups at the end of treatment in A431 tumor bearing model. Scale bar = 200 μm.

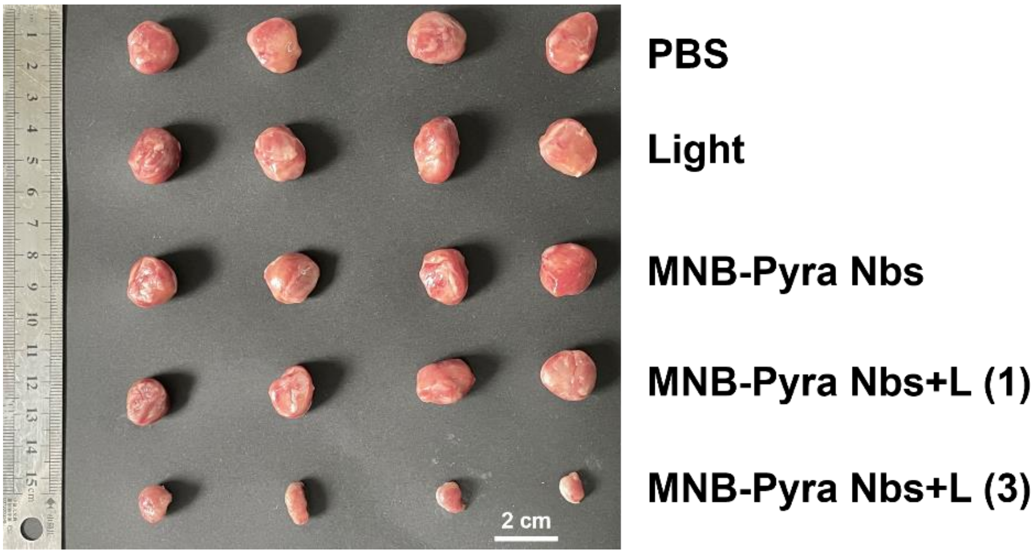

Supplementary Figure 31. Tumor picture at the end of experiment in different groups in Hela tumor bearing model. Scale bar = 2 cm.

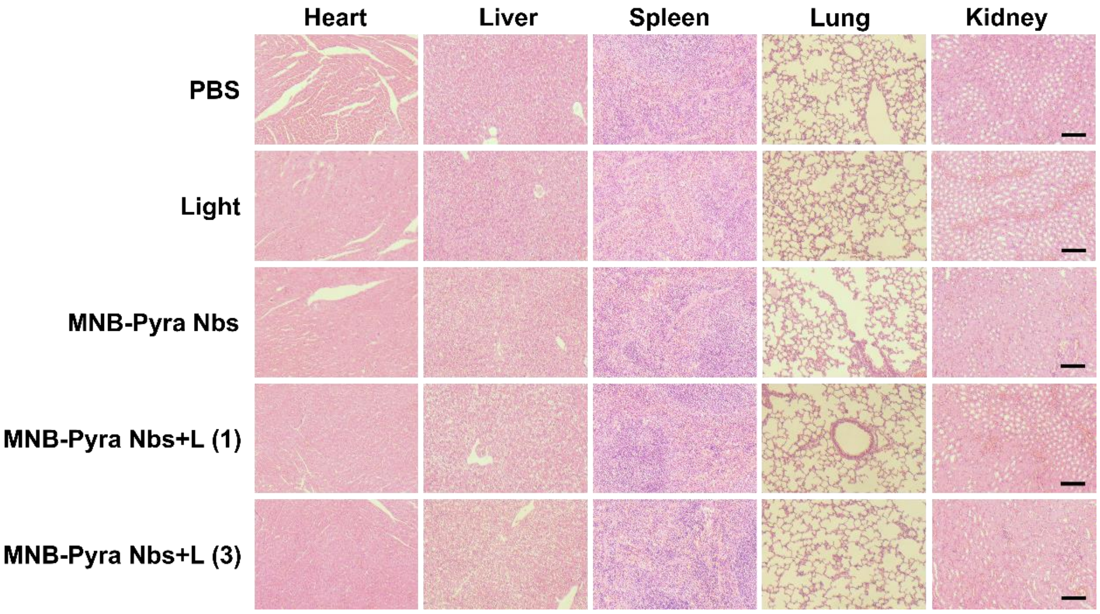

Supplementary Figure 32. H&E analysis of the major organs (heart, liver, spleen, lung, and kidney) tissues collected from mice in the different groups at the end of treatment in Hela tumor bearing model. Scale bar = 200 μm.

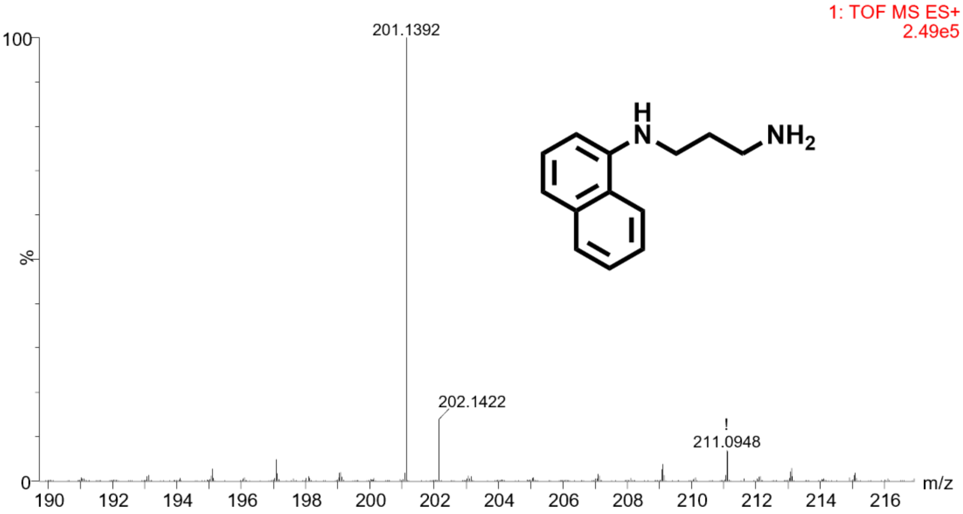

Supplementary Figure 33. HRMS spectrum of compound 1.

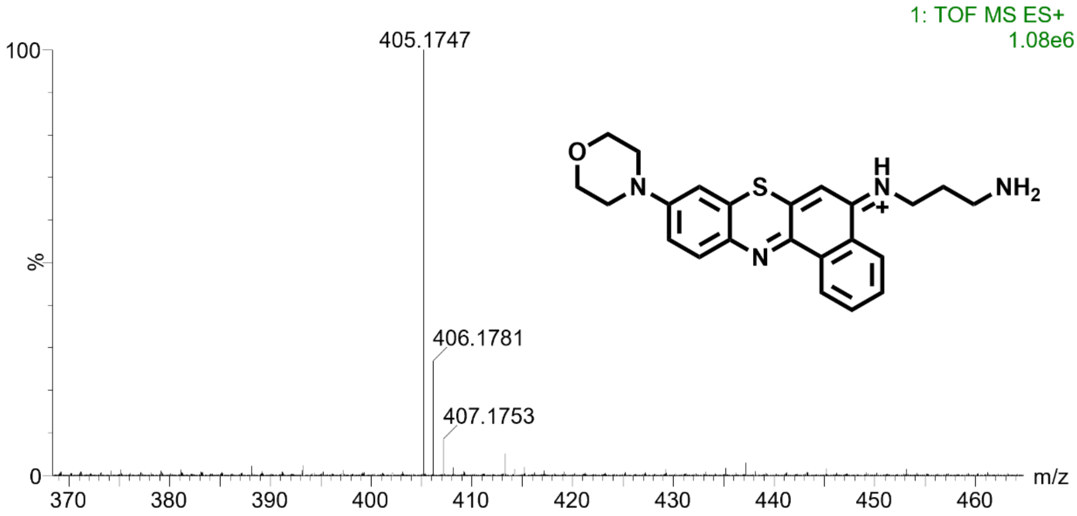

Supplementary Figure 34. HRMS spectrum of MNB.

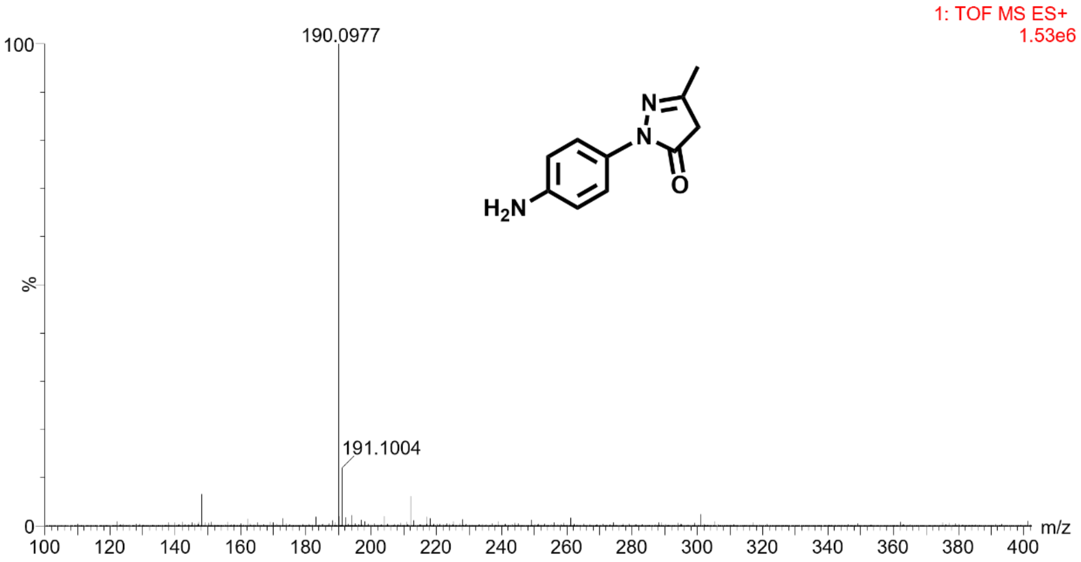

Supplementary Figure 35. HRMS spectrum of compound 3.

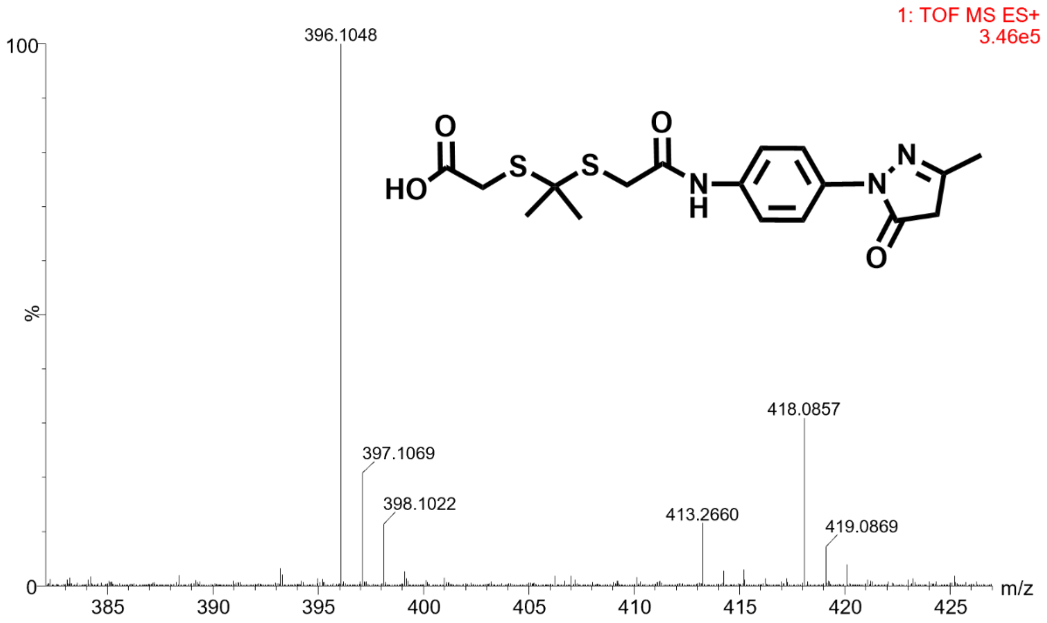

Supplementary Figure 36. HRMS spectrum of compound 4.

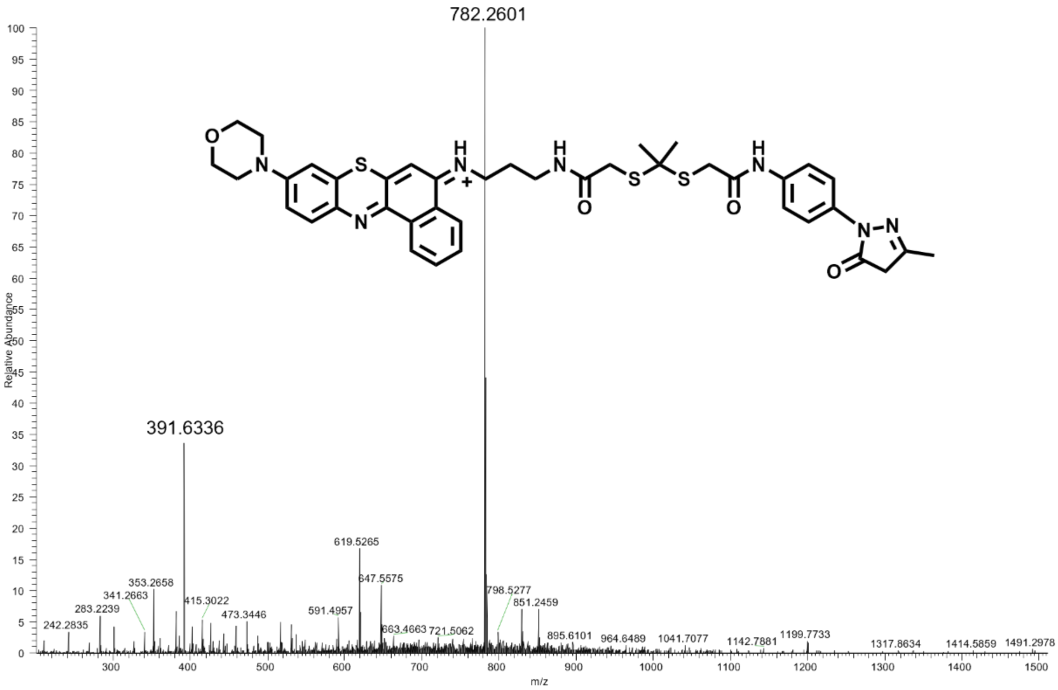

Supplementary Figure 37. HRMS spectrum of MNB-Pyra.

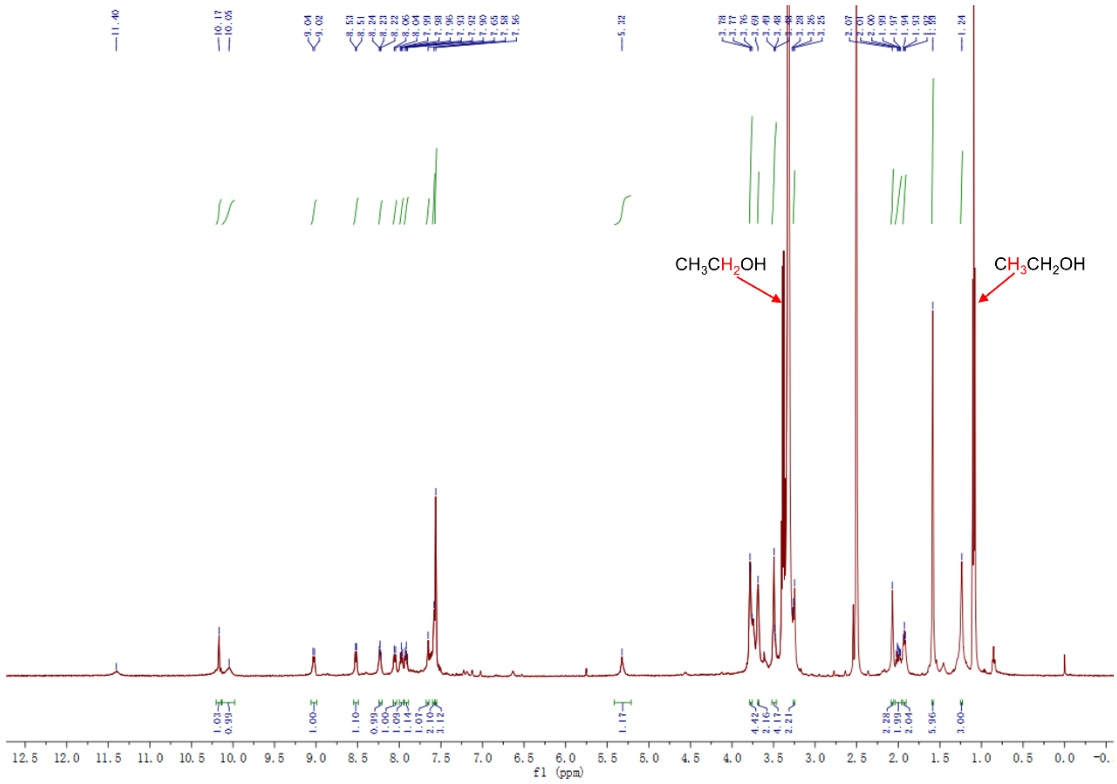

Supplementary Figure 38. ^{1}H NMR spectrum of MNB-Pyra.

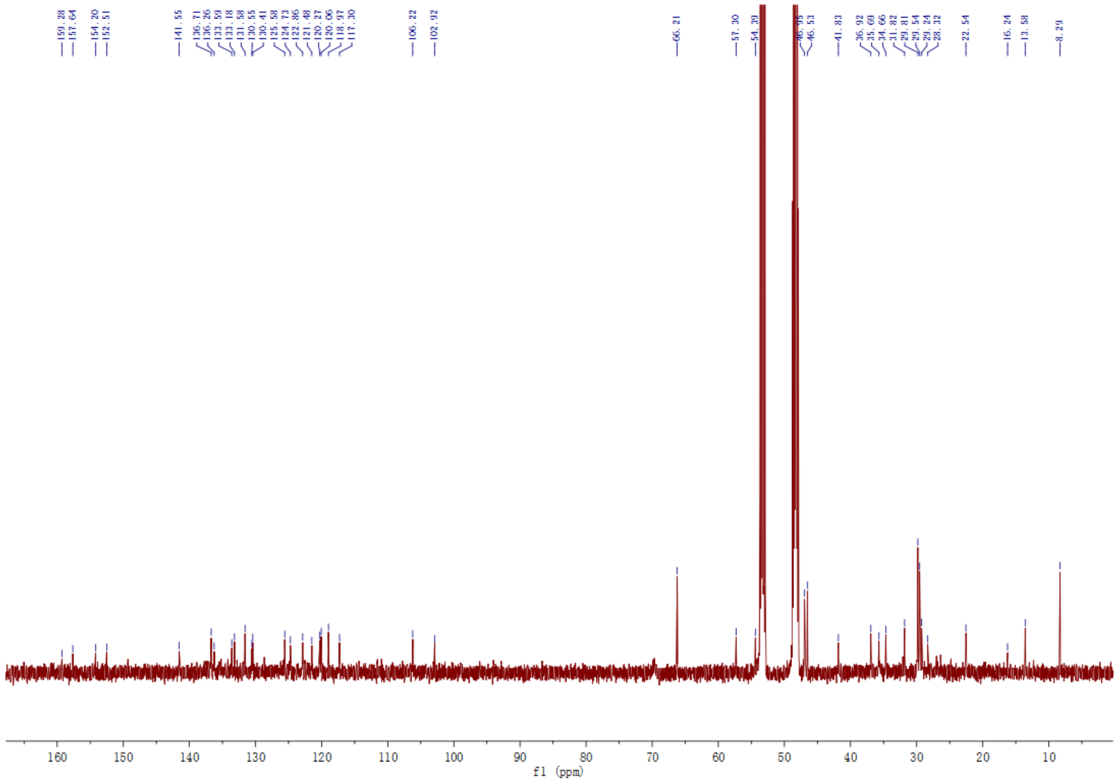

Supplementary Figure 39. ^{13}C NMR spectrum of MNB-Pyra.

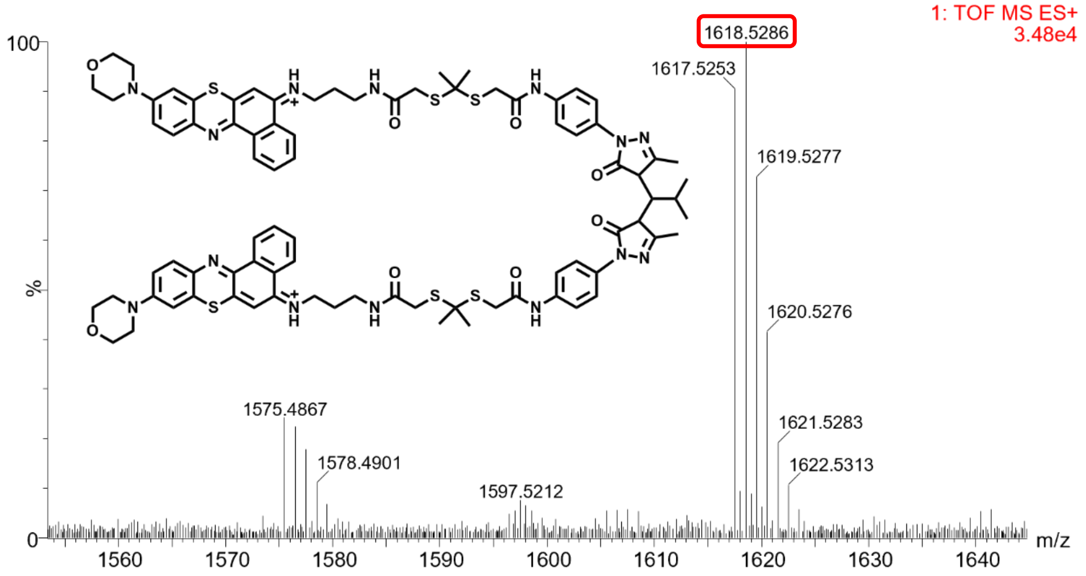

Supplementary Figure 40. HRMS spectrum of MNB-Pyra dimer.

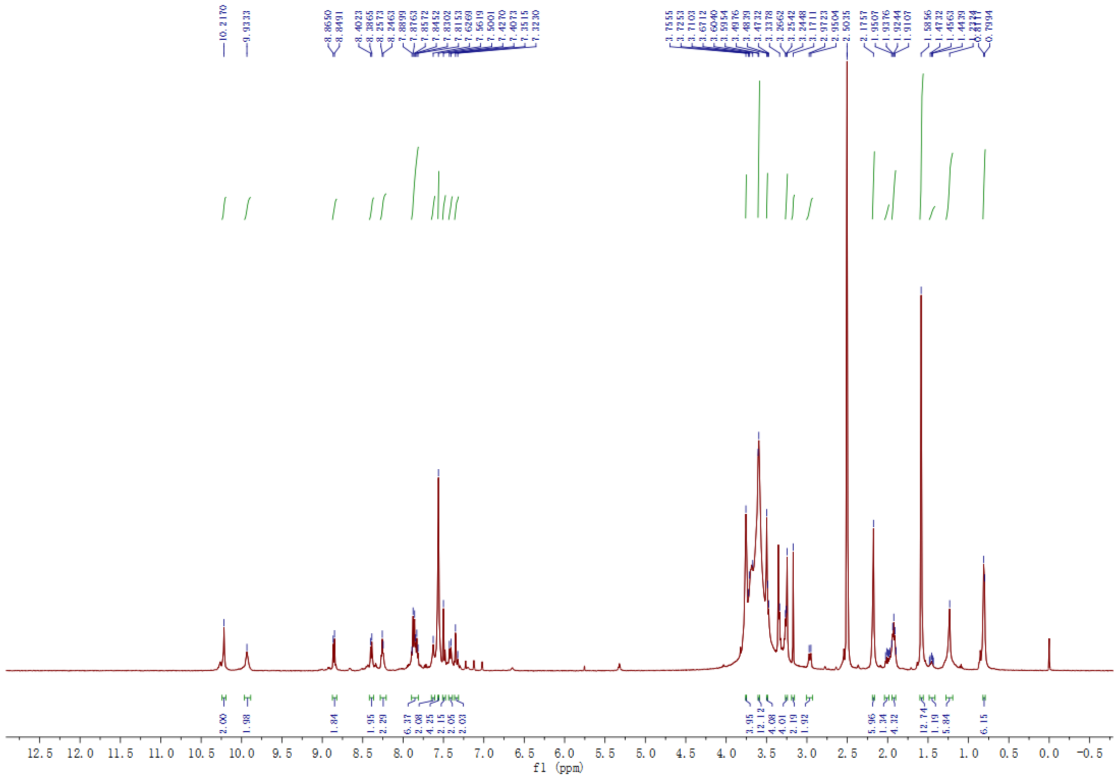

Supplementary Figure 41. ^{1}H NMR spectrum of MNB-Pyra dimer.

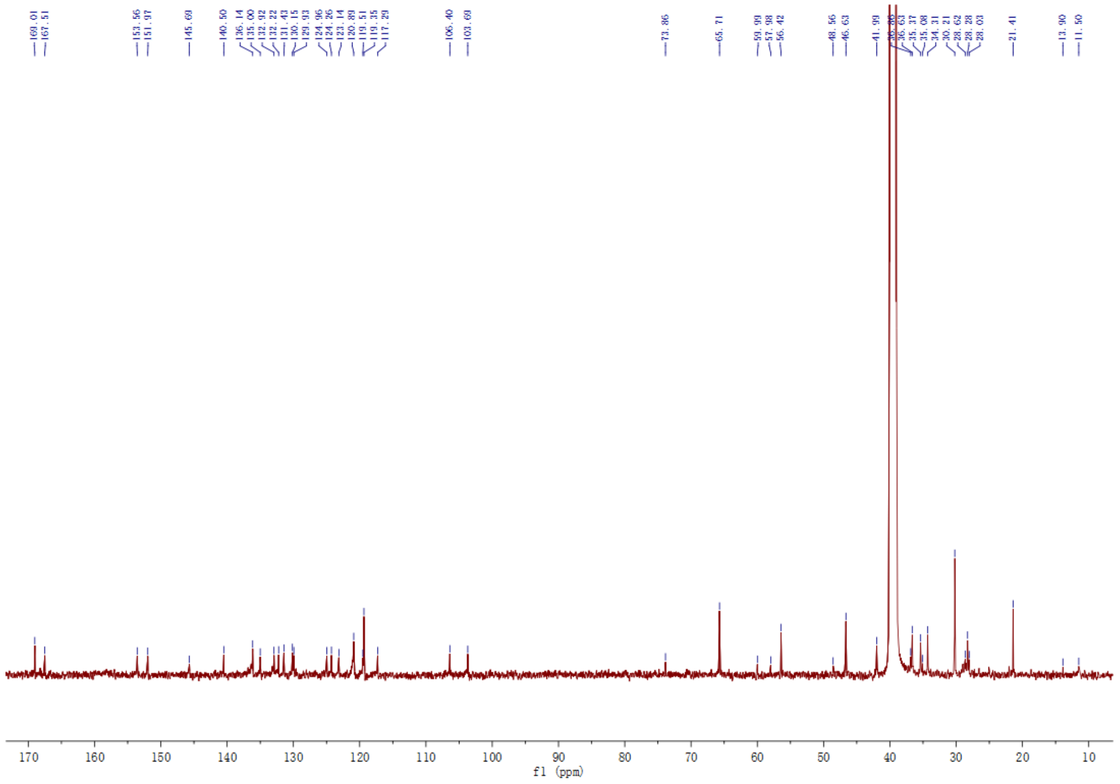

Supplementary Figure 42. ^{13}C NMR spectrum of MNB-Pyra dimer.
