# Supplementary material for: Self-reporting photodynamic nanobody conjugate for precise and sustainable large-volume tumor treatment
Source: Nat Commun. 2024 Aug 13;15:6935. doi: 10.1038/s41467-024-51253-5 (PMC11322375; doi:10.1038/s41467-024-51253-5)
Supplement: Supplementary file 3 — Reporting Summary [file 41467_2024_51253_MOESM3_ESM.pdf]

nature portfolio

Corresponding author(s): Jiangli Fan

Last updated by author(s): Jun 10, 2024

Reporting Summary

Nature Portfolio wishes to improve the reproducibility of the work that we publish. This form provides structure for consistency and transparency in reporting. For further information on Nature Portfolio policies, see our Editorial Policies and the Editorial Policy Checklist.

Statistics

For all statistical analyses, confirm that the following items are present in the figure legend, table legend, main text, or Methods section.

n/a	Confirmed	
☐	☒	The exact sample size (*n*) for each experimental group/condition, given as a discrete number and unit of measurement
☐	☒	A statement on whether measurements were taken from distinct samples or whether the same sample was measured repeatedly
☐	☒	The statistical test(s) used AND whether they are one- or two-sided<br>*Only common tests should be described solely by name; describe more complex techniques in the Methods section.*
☒	☐	A description of all covariates tested
☐	☒	A description of any assumptions or corrections, such as tests of normality and adjustment for multiple comparisons
☐	☒	A full description of the statistical parameters including central tendency (e.g. means) or other basic estimates (e.g. regression coefficient) AND variation (e.g. standard deviation) or associated estimates of uncertainty (e.g. confidence intervals)
☐	☒	For null hypothesis testing, the test statistic (e.g. *F*, *t*, *r*) with confidence intervals, effect sizes, degrees of freedom and *P* value noted<br>*Give P values as exact values whenever suitable.*
☒	☐	For Bayesian analysis, information on the choice of priors and Markov chain Monte Carlo settings
☒	☐	For hierarchical and complex designs, identification of the appropriate level for tests and full reporting of outcomes
☐	☒	Estimates of effect sizes (e.g. Cohen's *d*, Pearson's *r*), indicating how they were calculated

Our web collection on statistics for biologists contains articles on many of the points above.

Software and code

Policy information about availability of computer code

Data collection	The nuclear magnetic resonance of all compounds were detected by Bruker Avance II 500 MHz spectrometer; mass spectrometric data were obtained with electrospray ionization (EIS)-high resolution mass spectrometry (HRMS) analysis by Synapt G2-Si HDMS (Waters, USA); HRMS-High performance liquid chromatography(HPLC) was analyzed by LTQ Orbitrap XL (Thermo Scientific, USA); UV/vis spectra were performed on Lambda 60 UV-visible (UV-Vis) spectrophotometer (PerkinElmer, USA); fluorescence spectra were tested in a VAEIAN CARY Eclipse fluorescence spectrophotometer (Serial No. FL0812-M018); fluorescence images were obtained with a confocal laser scanning microscope (Olympus Fluoview FV3000); cytotoxicity assays were analyzed by Thermo Varioskan™ LUX multifunctional microplate reader (USA); flow cytometry was analyzed by Attune NxT Acoustic Focusing Cytometer; small animals' fluorescence imaging was carried out by NightOWL II LB983 living imaging system.
Data analysis	The resonance data was analyzed by MestRenova software; the mass spectrometric data of organic molecules were analyzed with Masslynx software (Version 4.1, Waters, USA); the mass spectrometric data of proteins were analyzed with Biopharma Finder (Version2.0, Thermo finisher, USA); the statistical analysis was performed with Origin software (Version 2021 , OriginLab, Northhampton, MA); the fluorescence images statistical analysis was performed with Olympus FV3000 software; the flow-cytometry data was analylzed on FlowJo software (V10, BDBiosciences, USA); Image J (1.8) was used to analyze the immunohistochemical results.PowerPoint and Figdraw were used to create some images.

For manuscripts utilizing custom algorithms or software that are central to the research but not yet described in published literature, software must be made available to editors and reviewers. We strongly encourage code deposition in a community repository (e.g. GitHub). See the Nature Portfolio guidelines for submitting code & software for further information.

Data

Policy information about availability of data

All manuscripts must include a data availability statement. This statement should provide the following information, where applicable:

- Accession codes, unique identifiers, or web links for publicly available datasets
- A description of any restrictions on data availability
- For clinical datasets or third party data, please ensure that the statement adheres to our policy

The authors declare that the data supporting the findings of this study are available within the article and its Supplementary Information files. Source data are provided with this paper. All remaining data can be found in the Article, Supplementary and Source Data files.

Research involving human participants, their data, or biological material

Policy information about studies with human participants or human data. See also policy information about sex, gender (identity/presentation), and sexual orientation and race, ethnicity and racism.

Reporting on sex and gender	No human study participants are involved in this manuscript.
Reporting on race, ethnicity, or other socially relevant groupings	No human study participants are involved in this manuscript.
Population characteristics	No human study participants are involved in this manuscript.
Recruitment	No human study participants are involved in this manuscript.
Ethics oversight	No human study participants are involved in this manuscript.

Note that full information on the approval of the study protocol must also be provided in the manuscript.

Field-specific reporting

Please select the one below that is the best fit for your research. If you are not sure, read the appropriate sections before making your selection.

☒ Life sciences ☐ Behavioural & social sciences ☐ Ecological, evolutionary & environmental sciences

For a reference copy of the document with all sections, see nature.com/documents/nr-reporting-summary-flat.pdf

Life sciences study design

All studies must disclose on these points even when the disclosure is negative.

Sample size	Pilot and published studies were used to determine the sample size. The mean and variance between groups was sufficient to determine if differences between groups were significant.
Data exclusions	No data exclusions was performed from the analyses in the manuscript.
Replication	The experiments were conducted at least twice independently, yielding consistent results. Replicates were reproducible.
Randomization	All biological experiments were randomized.
Blinding	Blinding was used in all biological experiments.

Reporting for specific materials, systems and methods

We require information from authors about some types of materials, experimental systems and methods used in many studies. Here, indicate whether each material, system or method listed is relevant to your study. If you are not sure if a list item applies to your research, read the appropriate section before selecting a response.

Materials & experimental systems

n/a	Involved in the study	
☐	☒	Antibodies
☐	☒	Eukaryotic cell lines
☒	☐	Palaeontology and archaeology
☐	☒	Animals and other organisms
☒	☐	Clinical data
☒	☐	Dual use research of concern
☒	☐	Plants

Methods

n/a	Involved in the study	
☒	☐	ChIP-seq
☐	☒	Flow cytometry
☒	☐	MRI-based neuroimaging

Antibodies

Antibodies used	In this work, a modified Anti-Human EGFR (7D12) nanobody was used for targeting the epidermal growth factor receptor of cells. This nanobody was prepared by the method in our previous work. DOI:10.1039/c9cy01856e; 10.1002/adfm.202103629.<br>7D12 (10 mg) nanobody was dissolved in MES buffer with a concentration of 30 μg/μL for the preparation of conjugate.<br>Protein sequences:<br>AEFQVKLEESGGGSVQTGGSLRLTCAASGRTSRSYGMGWFRQAPGKEREFVSGISWRGDSTGYADSVKGRFTISRDNAKNTVDLQMNSLKPEDTAIYYC AAAAGSAWYGTLYEYDYWGQGTQ VTVSSEPKTPKPQPQPQPQPQPNPTTEHHHHHHGGGGSLCTPSR
Validation	The molecular weight of this nanobody was verified by SDS-PAGE and mass spectrometry, and related validation could be find in the reference. DOI:10.1039/c9cy01856e; 10.1002/adfm.202103629.

Eukaryotic cell lines

Policy information about cell lines and Sex and Gender in Research

Cell line source(s)	Human epidermoid carcinoma cells (A431 cells; TCHu188) , human cervical carcinoma cells (HeLa cells; TCHu187), human non-small cell lung cancer cells (A549 cells; TCHu150) and mouse embryonic cells (NIH-3T3 cells; SCSP-5012) were purchased from the National Collection of Authenticated Cell Cultures (Shanghai, China).
Authentication	Short Tandem Repeat identification is correct according to report by supplier.
Mycoplasma contamination	No mycoplasma contamination was detected for all cell lines.
Commonly misidentified lines (See ICLAC register)	No cell line were misidentified.

Animals and other research organisms

Policy information about studies involving animals; ARRIVE guidelines recommended for reporting animal research, and Sex and Gender in Research

Laboratory animals	BALB/c nude mice, female, 5-6 weeks old. The mice were purchased from Beijing Vital River Laboratory Animal Technology Co., Ltd. Mice were housed in a specific pathogen-free (SPF) environment at the Laboratory Animal Center of Dalian University of Technology at a temperature of 22–26°C, relative humidity of 50–60%, and alternating light and dark every 12 h.
Wild animals	This study did not involve wild animals.
Reporting on sex	This study did not involve the reporting on sex.
Field-collected samples	This study did not involve samples collected from field.
Ethics oversight	Our research complies with all relevant ethical regulations. All animal experiments were approved by the animal research ethics committee of Dalian University of Technology. The approval number is DUT20210713.

Note that full information on the approval of the study protocol must also be provided in the manuscript.

Plants

Seed stocks	No plant study are involved in this manuscript.
Novel plant genotypes	No plant study are involved in this manuscript.
Authentication	No plant study are involved in this manuscript.

Flow Cytometry

Plots

Confirm that:

☒ The axis labels state the marker and fluorochrome used (e.g. CD4-FITC).

☒ The axis scales are clearly visible. Include numbers along axes only for bottom left plot of group (a 'group' is an analysis of identical markers).

☒ All plots are contour plots with outliers or pseudocolor plots.

☒ A numerical value for number of cells or percentage (with statistics) is provided.

Methodology

Sample preparation	Different cells (100000) planted in 6-well plate were incubated with 100 nM of MNB-Pyra Nbs for 2 h and treated with or without light irradiation (630 nm, 30mW/cm2, 20 min), followed by digestion and washing with PBS. Cells were then resuspended in PBS (200 μl) for test.
Instrument	Attune™ NxT flow cytometer (ThermoFisher Scientific)
Software	FlowJo_V10
Cell population abundance	At least 10000 cells were selected for fluorescence intensity statistics
Gating strategy	Cells without the incubation of MNB-Pyra Nbs were selected as negative control. The cells incubated with MNB-Pyra Nbs were positive experimental groups.

☐ Tick this box to confirm that a figure exemplifying the gating strategy is provided in the Supplementary Information.
